# Unique, Diverged, and Conserved Mitochondrial Functions Influencing Candida albicans Respiration

**DOI:** 10.1128/mBio.00300-19

**Published:** 2019-06-25

**Authors:** Nuo Sun, Rebecca S. Parrish, Richard A. Calderone, William A. Fonzi

**Affiliations:** a Department of Microbiology and Immunology, Georgetown University, Washington, DC, USA; Stockholm University; Leibniz Institute for Natural Product Research and Infection Biology—Hans Knoell Institute Jena (HKI)

**Keywords:** fungi, evolution, mitochondria, pathogens

## Abstract

Candida albicans is an opportunistic fungal pathogen of major clinical concern. The virulence of this pathogen is intimately intertwined with its metabolism. Mitochondria, which have a central metabolic role, have undergone many lineage-specific adaptations in association with their eukaryotic host. A screen for lineage-specific genes identified seven such genes specific to the CTG clade of fungi, of which C. albicans is a member. Each is required for respiratory growth and is integral to expression of complex I, III, or IV of the electron transport chain. Two genes, *NUO3* and *NUO4*, encode supernumerary subunits of complex I, whereas *NUE1* and *NUE2* have nonstructural roles in expression of complex I. Similarly, the other three genes have nonstructural roles in expression of complex III (*QCE1*) or complex IV (*COE1* and *COE2*). In addition to these novel additions, an alternative functional assignment was found for the mitochondrial protein encoded by *MNE1*. *MNE1* was required for complex I expression in C. albicans, whereas the distantly related Saccharomyces cerevisiae ortholog participates in expression of complex III. Phenotypic analysis of deletion mutants showed that fermentative metabolism is unable to support optimal growth rates or yields of C. albicans. However, yeast-hypha morphogenesis, an important virulence attribute, did not require respiratory metabolism under hypoxic conditions. The inability to respire also resulted in hypersensitivity to the antifungal fluconazole and in attenuated virulence in a Galleria mellonella infection model. The results show that lineage-specific adaptations have occurred in C. albicans mitochondria and highlight the significance of respiratory metabolism in the pathobiology of C. albicans.

## Abstract (precis)

Candida albicans is an opportunistic fungal pathogen of major clinical concern. The virulence of this pathogen is intimately intertwined with its metabolic behavior, and mitochondria have a central role in that metabolism. Mitochondria have undergone many evolutionary changes, which include lineage-specific adaptations in association with their eukaryotic host. Seven lineage-specific genes required for electron transport chain function were identified in the CTG clade of fungi, of which C. albicans is a member. Additionally, examination of several highly diverged orthologs encoding mitochondrial proteins demonstrated functional reassignment for one of these. Deficits imparted by deletion of these genes revealed the critical role of respiration in virulence attributes of the fungus and highlight important evolutionary adaptations in C. albicans metabolism.

## INTRODUCTION

The global burden of fungal infections is higher than is often appreciated (1). *Candida* spp., in particular, C. albicans, constitute a substantial share of this burden, with mortality rates of around 50% (1). C. albicans typically resides as a benign commensal of the skin and mucosal membranes, but immunological deficits or invasive medical procedures can alter the relationship between fungus and host, resulting in serious disseminated and invasive infections (2). The multifactorial pathogenicity of C. albicans relies on diverse processes, including yeast-hypha morphogenesis, phenotypic switching, biofilm formation, adherence, and secretion of hydrolases and toxins, among others (3–5). Metabolism and metabolic adaptations are central to driving these processes (5).

At the center of cellular metabolism are the mitochondria. They house and integrate multiple anabolic and catabolic functions involved in energy production, carbon, nitrogen, lipid and iron metabolism, and biosynthesis of amino acids, nucleic acids, and other cellular constituents. The integrity and function of mitochondria are essential to the virulence of C. albicans. Mutations affecting any one of a number of mitochondrial functions, including mitochondrial ribosome synthesis, mitochondrial transcription or genome maintenance, protein import, or functioning of the electron transport chain (ETC), result in avirulence (6–12). Avirulence is likely multifactorial, as mitochondrial mutations negatively impact growth rates and carbon source utilization (7, 8, 13), cell wall structure (7, 9, 14, 15), membrane composition (15), morphogenesis (7, 8, 13, 16, 17), and biofilm formation (18), as well as interactions with neutrophils (19) and macrophages (20). These observations emphasize the decisive role of mitochondria in the pathobiology of C. albicans (reviewed in reference 21). The significance of mitochondria extends well beyond a basic understanding of metabolism and virulence, as mitochondrial mutations have a meaningful impact on antifungal susceptibility (7, 11, 22).

Information about the structure and function of C. albicans mitochondria is limited, and, although mapping of basic information from other organisms is useful, mitochondria have experienced many adaptive evolutionary changes to accommodate the various habitats and the biology of their eukaryotic host (23–26). Even within the narrow scope of fungal phyla, notable differences occur (27–29). Comparison of C. albicans and Saccharomyces cerevisiae exemplifies the species-specific adaptations of mitochondria. Unlike S. cerevisiae, a Crabtree-positive yeast, C. albicans has been considered a Crabtree-negative species that maintains respiratory metabolism even in the presence of glucose (30, 31), although a recent report indicates that glucose can repress C. albicans respiration (32). This physiological difference is reflected in the dramatically different transcription networks controlling mitochondrial gene expression (33). The posttranscriptional control of mitochondrial protein synthesis has also undergone revision (15, 18), and C. albicans contains a complete ETC, while S. cerevisiae is devoid of complex I (NADH: ubiquinone oxidoreductase) (26, 28, 29).

A unique facet of complex I synthesis in C. albicans is the requirement for *GOA1*. *GOA1* was initially identified in a screen for mutants hypersensitive to oxidative stress (8). *GOA1* deletion mutants fail to make complex I, resulting in reduced respiration and multiple attendant deficits, including a reduced ability to form hyphae, cell wall alterations, and hypersensitivity to antifungals (8, 14, 19, 22, 34). The mutants also showed an enhanced sensitivity to killing by neutrophils, reduced immune recognition, and reduced virulence in a murine model of disseminated disease (8, 14, 19, 22, 34).

Interestingly, orthologs of *GOA1* are restricted to members of the “CTG clade” of fungi, whose members decode CTG codons as serine rather than leucine (35), suggesting that it represents a lineage-specific mitochondrial adaptation. Important issues are whether *GOA1* is a unique evolutionary adaptation or is representative of broader lineage-specific changes in mitochondrial function and what distinctive attributes might these genes bring to the pathobiology of C. albicans?

As a first step in addressing these issues, an *in silico* search was conducted for CTG clade-specific genes encoding putative mitochondrial proteins. Twenty-five potential genes of interest were identified, seven of which localized to mitochondria and were required for expression of various ETC complexes. Parallel analysis of three highly diverged mitochondrial genes with apparent orthologs in S. cerevisiae showed conservation of function for only two, *PET111* and *AEP1*, while *MNE1* demonstrated evolutionary reassignment. Regardless of the ETC complex affected, all of the respiratory mutants exhibited deficits in fitness, virulence, and antifungal susceptibility. The results revealed multiple lineage-specific adaptations unique to the CTG clade of fungi and suggest that additional adaptations may be found in the form of functional reassignment of mitochondrial proteins.

The data further emphasize the importance of mitochondrial function in the pathobiology of C. albicans.

## RESULTS

### *In silico* identification of putative clade-specific mitochondrial proteins.

Orthologs of *GOA1*, which is required for complex I function in C. albicans, are confined to the CTG clade of fungi (8). To assess the scope of clade-specific mitochondrial functions, an *in silico* screen was conducted. Clade-specific orthologs across eight species were identified and assessed for potential mitochondrial localization using three different algorithms, MitoPred (36), TargetP (37), and Predotar (38). Assessing multiple orthologs of slightly differing sequence was intended to eliminate fortuitous false positives, as all orthologs should embody the characteristics of a mitochondrial protein regardless of sequence variation. The use of multiple prediction algorithms provided a more rigorous assessment to address the low sensitivity of the prediction methods (39). The screen identified 25 genes of interest (Table 1). Importantly, the list included *GOA1*, validating the screening process. A total of 23 of the 25 genes (with the exceptions being *GOA1* and *FGR39*) were uncharacterized open reading frames (ORFs) encoding proteins of unknown function (Table 1). As evidenced here, several of these genes influenced expression of electron transport chain complex I, III, or IV and were the focus of this work. These included *NUO3* (orf19.1179, NADH:ubiquinone oxidoreductase), *NUO4* (orf19.5077), *NUE1* (orf19.2819, NADH:ubiquinone oxidoreductase expression), and *NUE2* (orf19.4467), which affect complex I; *QCE1* (orf19.6918, coenzyme Q:cytochrome *c* oxidoreductase expression), affecting complex III; and two genes affecting complex IV expression, *COE1* (orf19.1371, cytochrome *c* oxidase expression) and *COE2* (orf19.6566).

**TABLE 1:** Genes encoding putative mitochondrial proteins^*a*^

ORF identifier^*b*^	Gene name	Non-*Candida* ortholog	No. of CTG clade orthologs predicted by^*c*^:		
			MitoPred	TargetP	Predotar
**19.3818**	**GOA1**	**None**	**8**	**8**	**8**
**19.1179**	**NUO3**	**None**	**8**	**7**	**0**
**19.5077**	**NUO4**	**None**	**8**	**8**	**7**
**19.2819**	**NUE1**	**None**	**6**	**5**	**5**
**19.1371**	**COE1**	**None**	**8**	**7**	**8**
**19.6566**	**COE2**	**None**	**8**	**8**	**8**
**19.6918**	**QCE1**	**None**	**5**	**1**	**5**
**19.5607**		**None**	**8**	**8**	**8**
19.94		None	6	7	6
19.411		None	4	6	3
19.265		None	7	7	6
19.527		None	5	1	0
19.679	FGR39	None	8	8	8
19.935	AGA1	None	7	4	4
19.1287		None	5	2	0
19.1344		None	8	8	7
19.1748		None	4	8	3
19.1873		None	8	8	8
19.2650		None	8	8	8
19.3563		None	4	5	0
19.4553		None	5	4	0
19.4734		None	6	7	4
19.4795		None	7	7	8
19.4895		None	5	7	1
19.6853		None	5	5	5
**19.4467**	**NUE2**	**YALI0D21648**	**6**	**6**	**6**
**19.2513**	**MNE1**	**MNE1**	**7**	**7**	**7**
**19.230**	**PET111**	**PET111**	**6**	**5**	**5**
**19.102**	**AEP1**	**AEP1**	**5**	**4**	**4**

a Genes whose deletion resulted in failure to grow on glycerol are indicated with boldface characters. Those without an effect are indicated with lightface characters. NUO, NADH:ubiquinone oxidoreductase; NUE, NADH:ubiquinone oxidoreductase expression; COE, cytochrome *c* oxidase expression; QCE, coenzyme Q:cytochrome c oxidoreductase expression.

b Designations represent ORF identifiers according to the Candida Genome Database (http://www.candidagenome.org).

c Data represent numbers of CTG clade orthologs predicted to have a mitochondrial localization.

### Identification of genes affecting respiration.

As an initial screen for respiratory, defects homozygous deletion mutants were constructed for each gene of interest and tested for growth on nonfermentative carbon sources. Also included in the analysis were orf19.4467 (*NUE2*), which had only a single nonclade ortholog in Yarrowia lipolytica, and several genes distantly related to mitochondrial genes of S. cerevisiae, including *MNE1*, *PET111*, and *AEP1* (Table 1). These diverged proteins had ≤15% identity with the C. albicans ortholog, but the orthology data were supported by limited local synteny (40, 41). Their inclusion, with the objective of assessing whether these highly diverged orthologs retain analogous function, was motivated by the data showing gross differences between C. albicans and S. cerevisiae in mitochondrial composition and regulation (26, 29, 30, 33).

**FIG 1:**
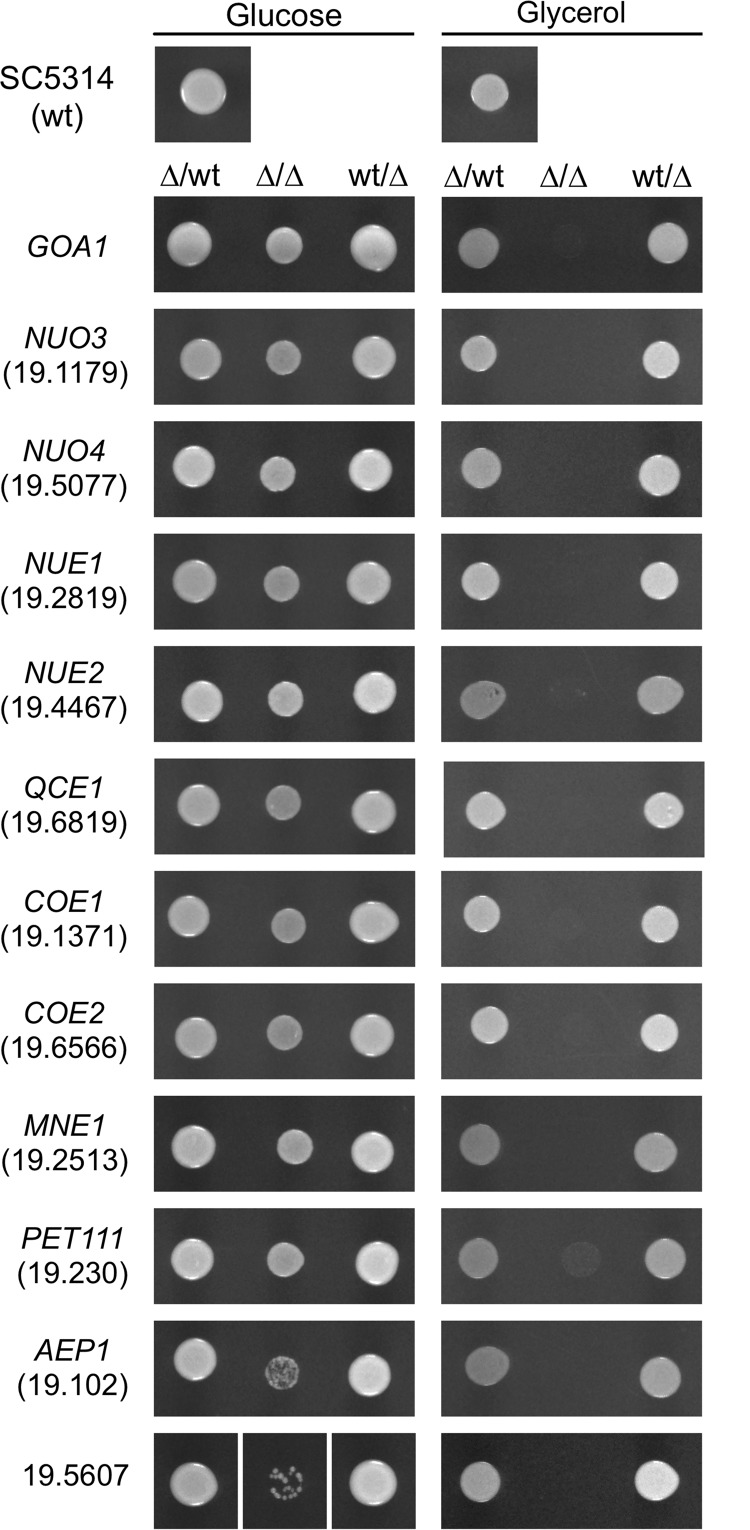
Phenotypes of mutants impaired in growth on glycerol. Approximately 2.5 × 10^4^ stationary-phase cells with the indicated genotype (heterozygous deletion (Δ/wild type [Δ/wt]), homozygous deletion [Δ/Δ], or complemented [wt/Δ]) were spotted on YPD or YPG and incubated at 30°C for 48 h (YPD) or 72 h (YPG), with the exception of the orf19.5607 null mutant, which was incubated 6 days on YPD. The pertinent gene name or ORF identifier or both are indicated on the left.

Homozygous deletion mutants were obtained for 24 of the 25 clade-specific genes. As expected, deletion of *GOA1* resulted in the inability to grow on glycerol. Among the other 23 deletion mutants, 7 were either unable to grow or showed severely limited growth on glycerol (Table 1; see also Fig. 1). Other mutants were deficient with respect to lacking *NUE2* or any one of the three S. cerevisiae orthologs (Table 1; see also Fig. 1). To rule out glycerol-specific metabolic defects, the mutants were tested for utilization of several nonfermentable carbon sources, including pyruvate, ethanol, and acetate. Consistent with an alteration in respiratory metabolism, the mutants were also unable to grow on these carbon sources. Complementation of the mutants with a wild-type copy of the cognate gene restored the ability to grow on nonfermentable carbon sources, demonstrating the link between genotype and phenotype (Fig. 1).

All of the putative respiratory mutants grew with glucose as the carbon source, but mutants lacking orf19.5607 showed extremely slow growth and a very low level of plating efficiency (Fig. 1). Because of their poor growth properties, these strains were not considered further and subsequent study focused on the other 10 mutants unable to grow on nonfermentative carbon sources.

### Respiration is reduced in the mutants.

The growth phenotype of the mutants suggested a deficiency in one or more respiratory pathways. C. albicans is capable of both cyanide-sensitive and cyanide-insensitive respiration, which represent the classic ETC and a short circuit of the ETC from ubiquinone to oxygen via alternative oxidases (42–44). The contribution of each was assessed using potassium cyanide (KCN) to inhibit cytochrome oxidase, complex IV, of the traditional electron transport chain and using salicylhydroxamic acid (SHAM) to inhibit alternative oxidase activity. As shown in Table 2, addition of KCN inhibited >95% of respiration in the control strain, in agreement with prior studies (42, 43). Subsequent addition of SHAM resulted in only a slight reduction in respiration.

**TABLE 2:** Effect of gene loss on respiration rate

Class	Strain	Genotype	Respiration rate^*a*^	% inhibition	
				KCN^*b*^	SHAM^*c*^
Wild type	SN152	Wild type	145.7 ± 6.4	95.6 ± 1.9	1.9 ± 0.8

I	Goa1ms-25	*goa1Δ*/*Δ*	51.7 ± 4.7	60.4 ± 2.6	29.4 ± 2.7
	mt1179-21	*nuo3Δ*/*Δ*	54.4 ± 5.6	61.9 ± 2.9	27.3 ± 3.4
	mt5077-21	*nuo4Δ*/*Δ*	55.1 ± 7.2	67.5 ± 3.2	22.9 ± 3.8
	mt2819-21	*nue1Δ*/*Δ*	52.7 ± 2.5	62.7 ± 4.0	26.4 ± 7.6
	mt4467-25	*nue2Δ*/*Δ*	51.3 ± 9.3	66.2 ± 5.4	18.8 ± 3.6
	mt2513-21	*mne1Δ*/*Δ*	49.8 ± 3.1	67.2 ± 3.4	24.2 ± 3.1

II	mt6918-24	*qce1Δ*/*Δ*	12.1 ± 0.7	14.6 ± 4.4	67.9 ± 4.5
	mt1371-21	*coe1Δ*/*Δ*	11.8 ± 0.8	14.9 ± 4.9	65.2 ± 4.7
	mt6566-21	*coe2Δ*/*Δ*	10.9 ± 1.6	14.6 ± 5.4	67.8 ± 5.8
	mt230-21	*pet111Δ*/*Δ*	10.8 ± 1.5	16.2 ± 1.7	64.2 ± 3.7

III	mt102-22	*aep1Δ*/*Δ*	8.2 ± 1.2	39.2 ± 3.6	14.9 ± 6.8

a Units represent nanomoles of O_2_/minute/milliliter·OD_595_ ± standard errors of the means (SEM) from at least 3 independent determinations.

b Data represent levels of inhibition of respiration by 1 mM potassium cyanide.

c Data represent levels of inhibition of respiration by 5 mM salicylhydroxamic acid.

The deletion mutants formed three classes based on O_2_ consumption rates and response to inhibitors. Class I mutants were similar to *goa1Δ*/*Δ* mutants. These retained only 35% of the wild-type respiratory capacity. Addition of KCN inhibited 60% to 65% of this activity, and addition of SHAM inhibited the majority of the remaining activity. This phenotype was conferred by deletion of *NUO3*, *NUO4*, *NUE1*, *NUE2*, or *MNE1* (Table 2). Approximately 70% of respiration in C. albicans is sensitive to rotenone, a specific inhibitor of complex I, and the remaining 30% is contributed by an alternative, rotenone-insensitive NADH-Q oxidoreductase (45). Rotenone treatment of the parental strain inhibited respiration by about 60% but had no measurable effect on class I mutants. Together, these results indicate that class I mutants are deficient in complex I and that residual respiration is due to the presence of the alternative, rotenone-insensitive NADH-Q oxidoreductase in conjunction with the SHAM-sensitive alternative oxidase.

The remaining five mutants formed classes II and III. These showed a more severe reduction in respiration, with O_2_ consumptions rates approximately 7% of control rates, and differed in their response to inhibitors. O_2_ utilization was inhibited only 15% by KCN but was inhibited 65% by SHAM. This indicates that approximately 99% of ETC-mediated respiration was eliminated in these mutants and that the bulk of oxygen consumption proceeded via the alternative oxidase. Deletion of *QCE1*, *COE1*, *COE2*, or *PET111* conferred this phenotype.

Class III consisted of a single mutant deleted of orf19.102, orthologous to S. cerevisiae *AEP1* (Sc*AEP1*). The rate of respiration was slightly less than that seen with the class II mutants and was inhibited about 40% by KCN, which translates into a 98% reduction in ETC activity. The remainder was SHAM sensitive. For all of the mutants, genetic rescue restored the rate and pattern of respiration to those seen with the wild-type control.

### Mutants lack various ETC complexes.

**FIG 2:**
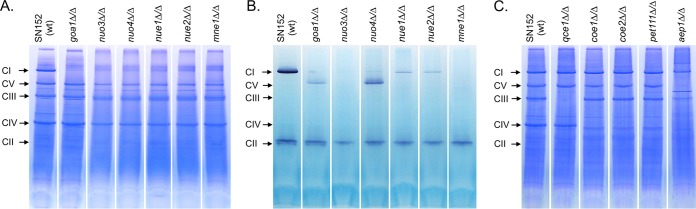
Blue native gel electrophoresis reveals loss of mitochondrial electron transport chain components. Purified mitochondria from the parental wild-type (SN152) and null mutant strains were subjected to detergent solubilization and fractionated on polyacrylamide gradient gels in the presence of Coomassie blue G250. (A) Samples of class I mutants. (B) Samples of class I mutants stained for NADH oxidase activity. The lower-molecular-weight oxidase activity presumably corresponds to the alternative, rotenone-insensitive NADH-Q oxidoreductase. Panel C shows class II and III mutants. The electrophoretic positions of complexes I, II, IV, and V are indicated on the right and were established by in-gel activity staining (87). The position of complex III was inferred from its relative size, since no activity stain is available. The relevant genotype is indicated above each lane. Comparable results were obtained in two or more mitochondrial preparations.

The deficits implied by respiratory measurements were directly supported by examination of ETC complexes (46, 47). All class I mutants exhibited partial or complete loss of complex I, consistent with loss of rotenone-sensitive respiration (Fig. 2A). Staining of the gels for NADH dehydrogenase activity showed greatly reduced amounts of intact complex I in *nue1Δ*/*Δ* and *nue2Δ*/*Δ* mutants, the presence of partial complexes in *goa1Δ*/*Δ* and *nuo4Δ*/*Δ* mutants, and a complete loss of activity in *nuo3Δ*/*Δ* and *mne1* mutants (Fig. 2B). There was no discernible loss of the other ETC complexes (Fig. 2A). Thus, *NUO3*, *NUO4*, *NUE1*, *NUE2*, and *MNE1* are required for proper expression of NADH:ubiquinone oxidoreductase (complex I).

Class II mutants exhibited one of two patterns. Deletion of *QCE1* resulted in loss of complex III with no observable effect on other ETC components (Fig. 2C). In contrast, mutants lacking *COE1*, *COE2*, or *PET111* were specifically devoid of complex IV (Fig. 2C) and no cytochrome oxidase activity could be detected in these mutants. These results are consistent with the essentially complete loss of KCN-sensitive respiration in these mutants. As with its respiratory profile, the class III *AEP1* null mutant was unique in its ETC component profile. The levels of complexes I and III were reduced, and complexes IV and V were absent (Fig. 2C).

### The genes encode mitochondrial proteins.

**FIG 3:**
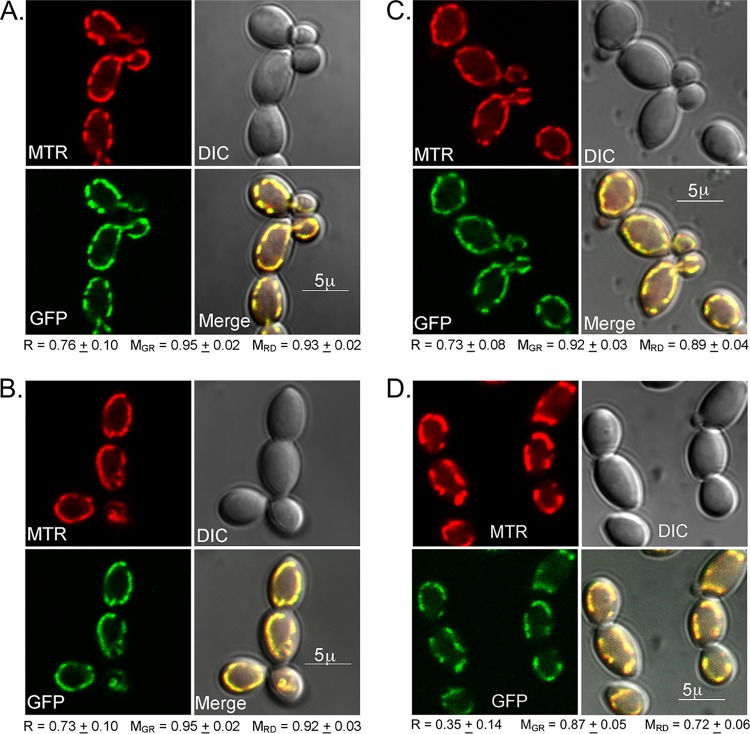
Colocalization analysis of mitochondrial proteins. Cells containing GFP fused to the gene of interest were stained with MitoTracker Red CMXRos, fixed, and examined by confocal microscopy. Images from the MitoTracker Red (MTR) channel and the GFP channel (GFP) and differential interference contrast (DIC) images are marked accordingly. Panels A to D show representative images from strains containing Cox8p-GFP, Nuo3p-GFP, Nuo4p-GFP, and AEP1p-GFP, respectively. Above-threshold Pearson’s correlation coefficient (R), green-channel Manders coefficient (M_GR_), and red-channel Manders coefficient (M_RD_) data are indicated below each image set. The values were determined as described by Costes et al. (51) and represent averages of results from at least 30 cells. The *P* value from the Costes significance test was 1.0 in each case. The intensity of the GFP signal was significantly less than that of the MitoTracker Red signal and was enhanced in the images to better visualize the correspondence.

The genes of interest were identified based on *in silico* predictions of mitochondrial localization, and these predictions were supported by confocal microscopy and cell fractionation studies. Green fluorescent protein (GFP) fusions were constructed and integrated into the wild-type allele of the corresponding heterozygous deletion mutant. All of the fusion genes allowed growth on glycerol, showing that the fusion proteins were functional. Mitochondria were labeled with the mitochondrion-specific dye MitoTracker Red CMXRos (48) and examined for colocalization of the dye and GFP signal. Cytochrome oxidase subunit Cox8p was fused with GFP and examined as a positive control (49, 50). As shown in Fig. 3A, there was a clear visual correspondence between MitoTracker staining and the Cox8-GFP signal. This was substantiated by quantitative analysis of the images (51). A statistically significant Pearson’s correlation coefficient and essentially identical Manders coefficients for all of the fluorescence channels (Fig. 3A) fully support the visual correlation. GFP fused to Nuo3, Nuo4, or Aep1 similarly showed a strong correspondence between dye and GFP signals (Fig. 3B, C, and D). For the remaining eight proteins, the GFP signal was not significantly above the background fluorescence of mitochondria. This was true of the Goa1-GFP fusion constructed in these studies, as well as the Goa1p-GFP strain previously examined (8). Fixed and unfixed cells yielded similar results.

**FIG 4:**
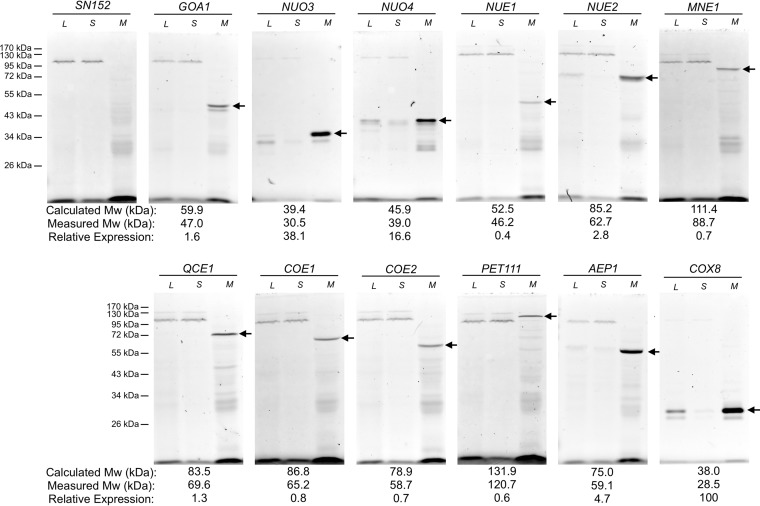
The respiratory proteins are enriched in mitochondria. Total cell lysates (L), mitochondrion-depleted supernatants (S), and mitochondria (M) were prepared from GFP-tagged strains and fractionated on SDS gels. An inverted fluorescence image of the gels is shown. The GFP-tagged gene is indicated above each lane set, and the mitochondrially enriched fluorescent protein is indicated by the arrow. The electrophoretic position of protein size markers is indicated on the left. The theoretical *M*_w_ of the fusion protein, the measured *M*_w_, and relative expression levels are indicated below each panel. Expression data are relative to that of Cox8-GFP and represent averages of results from two independent determinations.

As an alternative to direct visualization, the distribution of GFP-tagged proteins in various cell fractions was examined. As seen in Fig. 4, each strain contained a fluorescent protein that was strongly enriched in the mitochondrial fraction. The proteins roughly approximated the sizes calculated in the gene-based predictions but, as expected, were somewhat smaller since the samples were not fully denatured in order to preserve GFP fluorescence (52) and since mitochondrial proteins undergo signal cleavage postimport (53). Quantitation of the fluorescence intensities indicated an approximately 250-fold range in expression levels. Those proteins that were not detectable by confocal microscopy were expressed at <3% of the level of Cox8-GFP, but their enrichment in the mitochondrial fraction suggests that they too were localized to this organelle (Fig. 4).

**FIG 5:**
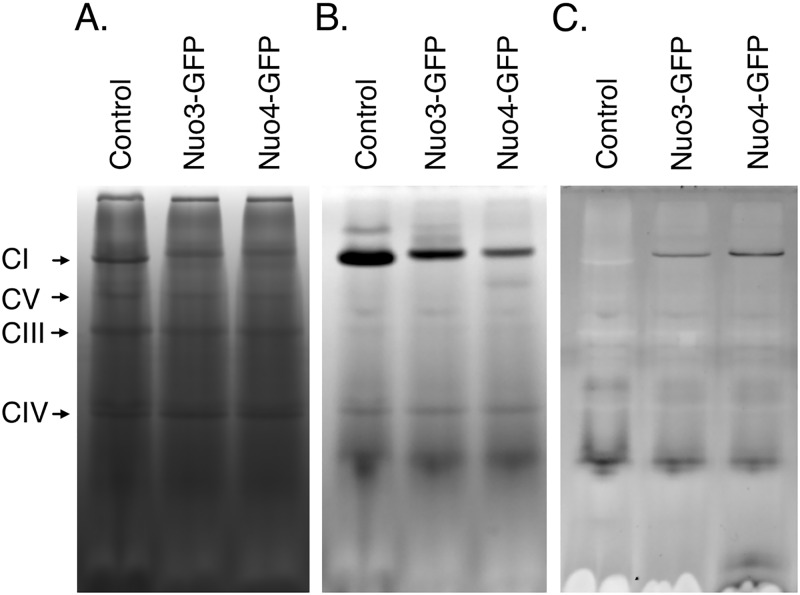
*NUO3* and *NUO4* encode subunits of complex I. Purified mitochondria from a wild-type strain or from strains in which GFP was fused to *NUO3* or *NUO4* were fractionated on blue native gels. (A) Protein staining. (B) NADH dehydrogenase activity. (C) Inverted fluorescent image. The relevant GFP fusion is indicated above each lane, and the position of each ETC complex is indicated on the left.

To assess whether these proteins have a structural role in the affected complexes, purified mitochondria of the GFP-tagged strains were fractionated on blue native gels. For both *NUO3* and *NUO4* GFP-tagged strains, complex I acquired fluorescence (Fig. 5). Furthermore, consistent with incorporation of GFP into the complex, complex I of these strains was slightly increased in size (Fig. 5). On the basis of their association with complex I, and consistent with the nomenclature of other complex I subunits, the corresponding genes were named *NUO3* and *NUO4* for (NADH:ubiquinone oxidoreductase). None of the other strains showed fluorescence associated with an ETC component. As the other genes did not appear to encode components of the electron transport chain complexes and were expressed at much lower levels, they apparently encode factors required for expression of the various complexes. Thus, orf19.2819 and orf19.4467 were designated *NUE1* and *NUE2*, respectively, reflecting their requirement for complex I expression (NADH:ubiquinone oxidoreductase expression). Similarly, the orf19.6918 deletion mutant lacked complex III and the gene was designated *QCE1* (coenzyme Q:cytochrome *c* oxidoreductase expression). ORFs 19.1371 and 19.6566 were designated *COE1* and *COE2* (cytochrome oxidase expression), recognizing their role in cytochrome oxidase (complex IV) expression.

### Growth rates and yields are reduced in respiratory mutants.

The relative contributions of fermentation and respiration to C. albicans metabolism are presently unclear (54, 55). Having an assemblage of mutants with various degrees of respiratory capacity provided the opportunity to assess fermentative contributions to growth. In synthetic complete (SC) medium, the rate of growth of complex I mutants was comparable to that of a wild-type strain despite their 65% reduction in respiration (Table 3). However, a 20% to 25% reduction in growth rate was seen when respiration via the ETC was eliminated, as in the mutants lacking complex III or IV. In synthetic defined (SD) medium, which imposes the additional metabolic burden of amino acid biosynthesis, growth rates were reduced 50% in complex I mutants and 75% in the other strains. Growth yields were also compromised. In SC medium, despite maintaining maximal growth rates, complex I mutants exhibited a 60% to 64% reduction in growth yield (Table 3). Mutants with a complete loss of KCN-sensitive respiration showed a 71% to 77% reduction (Table 3). Thus, fermentation alone cannot support maximal growth rates or yields.

**TABLE 3:** Growth rates and yield of mitochondrial mutants

Strain	Genotype	Defective complex	Doubling time (h)^*a*^		Growth yield (%)^*b*^
			SC medium	SD medium	
SC5314	Wild type	None	1.50 + 0.05	1.80 + 0.08	100
goa1ms.41	*goa1Δ*/*Δ*	Complex I	1.46 + 0.03	2.89 + 0.16	36 + 2
mt1179.41	*nuo3Δ*/*Δ*	Complex I	1.36 + 0.04	2.86 + 0.12	37 + 2
mt5077.41	*nuo4Δ*/*Δ*	Complex I	1.43 + 0.04	2.69 + 0.16	36 + 3
mt2819.41	*nue1Δ*/*Δ*	Complex I	1.44 + 0.04	2.82 + 0.13	36 + 2
mt4467.41	*nue2Δ*/*Δ*	Complex I	1.44 + 0.03	2.57 + 0.14	40 + 1
mt2513.41	*mne1Δ*/*Δ*	Complex I	1.44 + 0.04	2.76 + 0.08	36 + 3
mt6918.41	*qce1Δ*/*Δ*	Complex III	1.90 + 0.07	3.23 + 0.16	29 + 1
mt1371.41	*coe1Δ*/*Δ*	Complex IV	1.79 + 0.02	3.24 + 0.11	26 + 1
mt6566.41	*coe2Δ*/*Δ*	Complex IV	1.85 + 0.11	3.12 + 0.15	27 + 2
mt230.41	*pet111Δ*/*Δ*	Complex IV	1.82 + 0.02	3.13 + 0.18	29 + 3
mt102.41	*aep1Δ*/*Δ*	Multiple	1.87 + 0.03	3.28 + 0.12	23 + 2

a Doubling times are expressed as averages ± SEM of results from at least four independent determinations.

b Growth yield was determined on SC medium with 0.5% glucose. At that concentration, the yield was a function of the level of glucose. Results represent averages from three determinations ± SEM.

### Influence of respiratory mutations on filamentation varied with the inducing conditions.

The relationship of respiration to morphogenesis has been examined for over 40 years, with reduced respiration reported as promoting the yeast-to-hypha transition in some studies (56–58) and blocking the process in others (8, 10, 16, 59, 60). Hence, the panel of mutants was examined for their ability to filament under various conditions.

**FIG 6:**
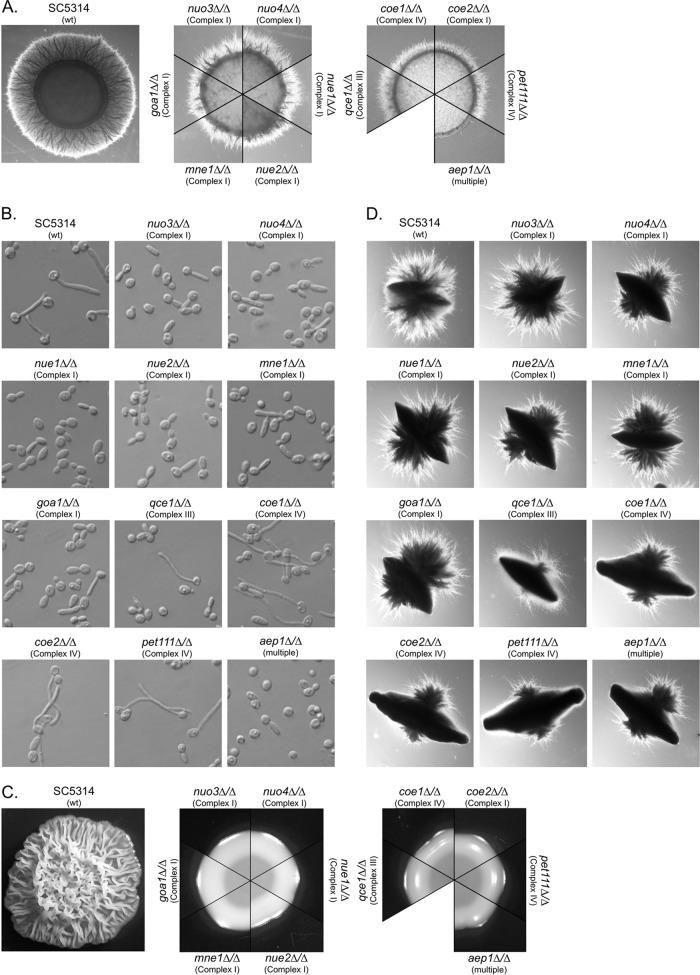
The effect of respiratory deficiency on filamentation varies with the inducing environment. (A) Filamentation response of cells spotted on medium 199 agar and incubated 72 h at 37°C. The wild-type colony and a representative segment of each mutant colony are shown. (B) Morphological response of cells incubated 3 h in medium 199 broth. (C) Filamentation response of cells spotted on YPD-plus-10% serum agar and incubated 48 h at 37°C. The wild-type colony and a representative segment of each mutant colony are shown. (D) Filamentation response of cells embedded in YPD-plus-10% serum agar and incubated 72 h at 37°C. A representative colony of each strain is shown. The relevant genotype and affected respiratory complex are indicated for each strain.

On medium 199 agar, wild-type cells formed a halo of abundant agar-invading hyphae around the colony center (Fig. 6A). Mutants defective in complex I were still capable of forming hyphae, but these were less abundant and shorter in length than those seen with the wild-type strain (Fig. 6A). Mutants lacking complex III or IV were similarly (or perhaps slightly more) compromised. The *aep1*Δ mutant, in contrast, was nearly devoid of hyphae. Kinetic analysis of the process showed that filamentation by the mutants was delayed in onset and that hyphal extension occurred at reduced rates (see Fig. S1 in the supplemental material). Under conditions of testing performed in the same media, but in broth suspension, a slightly different pattern was observed. Whereas the wild-type strain formed germ tubes and hyphae, the mutants lacking complex I were restricted to pseudohyphae and yeast (Fig. 6B). But, surprisingly, germ tubes and true hyphae were formed by complex III and IV mutants, although at lower levels of abundance than were seen with the wild type. Again, the *aep1Δ* mutant was unable to form hyphae. Extending the incubation time did not result in germ tube formation by complex I mutants.

10.1128/mBio.00300-19.2 FIG S1 (A) Kinetics of hyphal growth on medium 199 agar. Spot colonies on medium 199 agar were imaged at 24-h intervals, and the width of the hyphal halo surrounding each colony was measured. Results from experiments performed with a representative complex I mutant (*nuo3Δ*/*nuo3Δ*), a complex IV mutant (*coe1Δ*/*coeΔ*), and the complex III mutant (*qce1Δ*/*qce1Δ*) are shown. Measurements represent averages of results from at least three independent determinations. Error bars indicate standard errors of the means. Download FIG S1, TIF file, 1.8 MB. Copyright © 2019 Sun et al. 2019 Sun et al. This content is distributed under the terms of the Creative Commons Attribution 4.0 International license.

On yeast extract-peptone-dextrose (YPD)-plus-10% serum agar or YNBAGNP (1.5% agar, 0.67% yeast nitrogen base [YNB] medium with ammonium sulfate, 10 mM dextrose, 5 mM GlcNAc, 2% [wt/vol] Casamino acids, 25 mM potassium phosphate buffer) agar (59, 60), wild-type cells form a highly wrinkled, biofilm-like colony consisting of an extensive hyphal network, yeast, and pseudohyphae (Fig. 6C; see also Fig. S1). The respiratory mutants, however, formed flat, smooth colonies (Fig. 6C). The colony interiors predominantly contained yeast with only occasional hyphae, unlike the tangled mesh of hyphae seen in the wild-type strain (Fig. S2). Extending the incubation time several days did not result in formation of wrinkled colonies.

10.1128/mBio.00300-19.3 FIG S2 Respiratory deficiencies limit hypha formation under conditions that induce biofilm-like colonies. (A) Morphology of cells from within the colony dome of strains incubated 48 h at 37°C on YPD-plus-10% serum agar. Cells were imaged at ×40 magnification. (B) Filamentation response of cells spotted on YNBAGN agar and incubated 48 h at 37°C. The wild-type colony and a representative segment of each mutant colony are shown. The relevant genotype and affected respiratory complex are indicated for each strain. Download FIG S2, TIF file, 2.9 MB. Copyright © 2019 Sun et al. 2019 Sun et al. This content is distributed under the terms of the Creative Commons Attribution 4.0 International license.

Reduced oxygen tension has been reported as having either a negative or positive effect on filamentation (57–59, 61). The effect of embedding the cells in YPD-plus-serum agar was tested to examine the influence of reduced oxygen tension. Although the mutants failed to filament on the surface of this medium, all formed hyphae when embedded. The extent of filamentation of complex I mutants was difficult to distinguish from the level shown by the wild-type strain, while complex III and IV mutants were compromised only slightly (Fig. 6D). Complementation of the mutations restored a wild-type phenotype under all conditions. Together, these results indicate that respiration is not essential to filamentation and that its influence is dependent on the inducing environment.

### Mutants are hypersensitive to fluconazole.

**FIG 7:**
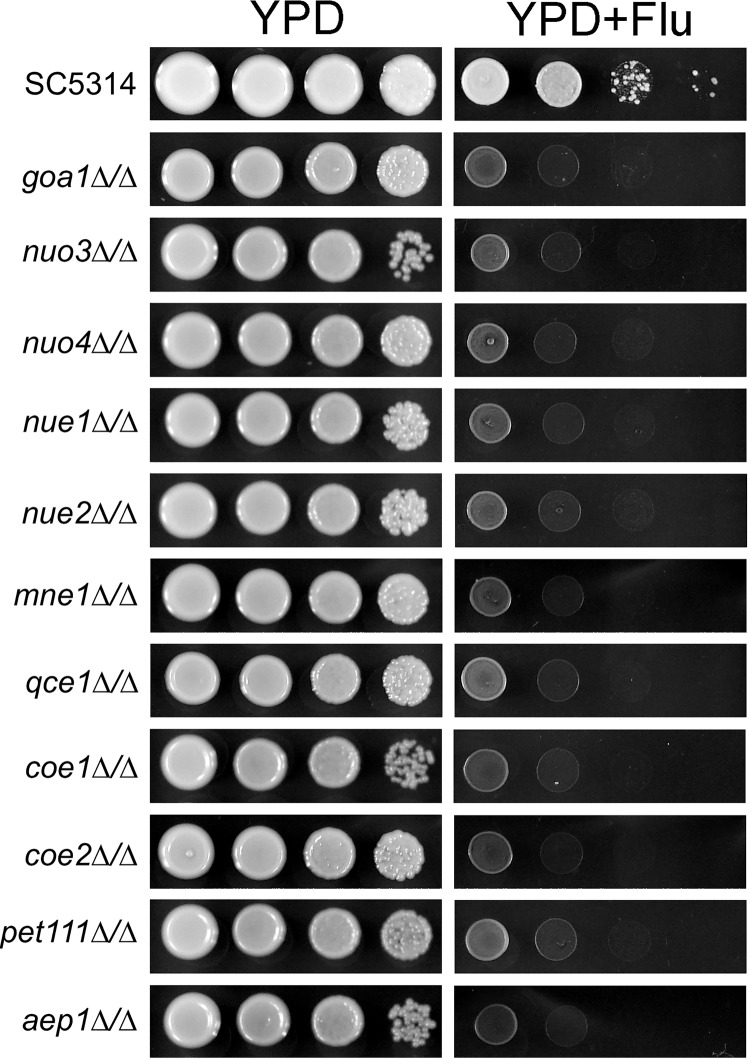
Respiratory mutants are hypersensitive to fluconazole. Serial dilutions of strains with the indicated genotype were spotted on YPD or YPD with fluconazole (FLU; 8 μg/ml) and incubated 48 h at 30°C.

Pharmacological inhibition of complexes I, III, IV, and V causes hypersensitivity to fluconazole (22). Fluconazole sensitivity of the respiratory mutants was examined to provide genetic validation of these results. As seen in Fig. 7, all of the respiratory mutants showed hypersensitivity to fluconazole, and the hypersensitivity was lost upon genetic rescue. This further validates the relationship between respiration and fluconazole sensitivity.

### Virulence is attenuated in the G. mellonella infection model.

**FIG 8:**
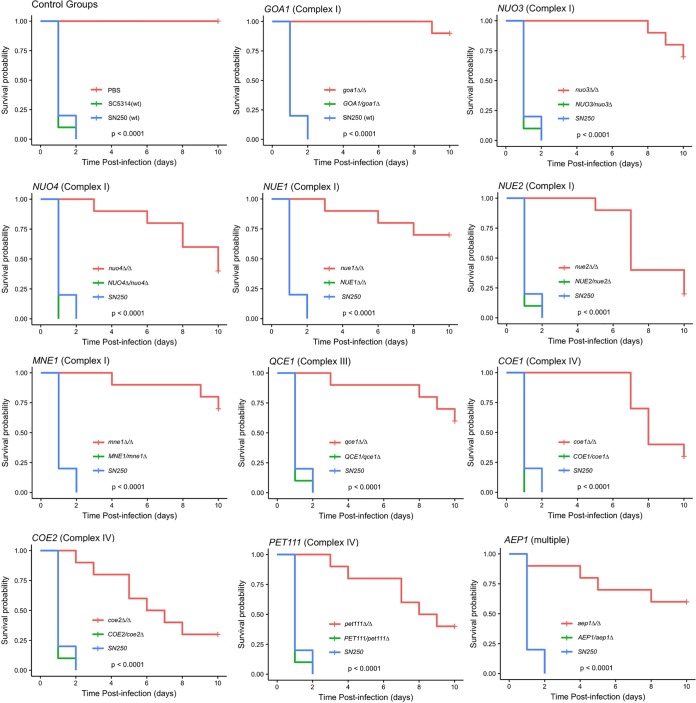
Virulence of respiratory mutants is attenuated in G. mellonella. The virulence of each gene deletion strain and that of the corresponding rescued strain were tested in the G. mellonella infection model. Kaplan-Meier survival curves and log-rank statistics are shown. The relevant gene is indicated above each plot.

Various C. albicans mitochondrial mutants were shown to be attenuated in a mouse model of disseminated disease (6–11). However, as shown by the filamentation phenotypes, the consequence of respiratory deficiency varies with the growth environment. Therefore, the virulence of the respiratory mutants was assessed in an alternative infection model represented by the greater wax moth Galleria mellonella (62). Mutants lacking *GOA1* are attenuated in mice (8) and were strongly attenuated in G. mellonella as well (Fig. 8). Likewise, virulence of all of the other respiratory mutants was significantly attenuated in this model (Fig. 8). The results indicate that respiration is critical to virulence in the G. mellonella model, as in the mouse model.

## DISCUSSION

Mitochondria have a central and critical role in the metabolism and pathobiology of C. albicans (21). Mitochondria perform multiple, diverse processes within the eukaryotic host cell and have evolved in composition and function among various phylogenetic lineages (25, 63). The results of this study identify a number of lineage-specific changes in composition and expression of the ETC in the CTG clade of fungi. In addition to *GOA1*, 24 putative CTG clade-specific genes were identified, seven of which have essential roles in respiration. The current analysis does not distinguish whether these are newly invented genes or orthologs of other fungal proteins that have diverged beyond recognition. Overall, these proteins represent a relatively small fraction of the mitochondrial proteome, which consists of about 1,000 proteins in yeast and humans (64, 65), but their importance is demonstrated by their critical role in mitochondrial function. The other 17 genes may function in other mitochondrial processes or may represent false positives, as the sensitivity of the prediction algorithms is around only 40% (39).

The biochemical role of these seven proteins in ETC expression remains to be determined. Two proteins, Nuo3 and Nuo4, were expressed at levels similar to that of Cox8, a subunit of complex IV. This observation, in conjunction with their physical association with complex I during electrophoresis, suggests that they are subunits of the complex. Eukaryotic complex I consists of a core of 14 proteins derived from the bacterial ancestor of mitochondria supplemented with various numbers of supernumerary subunits, e.g., 40 in Y. lipolytica (66) and 46 in mammals (26). Evolutionary addition and deletion of subunits have occurred broadly across phylogenetic lineages, including fungi (26, 28, 29). Nuo3 and Nuo4 appear to represent another example of evolutionary adaptation of complex I that is specific to the CTG clade. The function of the majority of supernumerary subunits of complex I has yet to be defined (63).

The other respiratory proteins were much less abundant than Nuo3 and Nuo4 and were not observed to associate with any of the ETC complexes. These proteins may participate in expression and assembly of the respiratory complexes, an intricate process requiring proteins that facilitate transcription, mRNA processing, and translation and posttranslational processing (67, 68) of component proteins. Alternatively, these proteins may be minor, nonstoichiometric subunits of the ETC complexes whose association was not apparent due to their low abundance. Goa1 was among these less abundant proteins, and the signal intensity of the GFP-tagged protein was not sufficiently above mitochondrial background fluorescence to localize it to the mitochondria. A previous report of differential localization of Goa1 appears to indicate a preparation artifact (8).

Beyond lineage-specific adaptations, the sharply different levels of regulation of respiration by C. albicans and S. cerevisiae (30, 33) might suggest that considerable functional divergence of orthologous proteins has occurred. Examination of three highly diverged orthologs, *MNE1*, *PET111*, and *AEP1*, suggests that this is partly true. A striking outcome was the absence of complex I in the *mne1Δ*/*Δ* mutant. In S. cerevisiae, Mne1 facilitates excision of the aI5β intron, one of seven introns in the *COX1* mRNA, and mutants lacking *MNE1* are devoid of complex IV (69). In contrast, C. albicans *mne1Δ*/*Δ* mutants had normal levels of complex IV but were devoid of complex I. Interestingly, the seven mitochondrially encoded subunits of complex I contain no introns, suggesting that C. albicans Mne1 (CaMne1) lacks even an intron splicing function. Given that complex I is an ancestral component of the ETC that was subsequently lost in S. cerevisiae and other Saccharomycetales (26, 28, 29), *CaMNE1* likely retains the ancestral function and functional reassignment occurred in S. cerevisiae.

Unlike that of *MNE1*, the function of *PET111* and *AEP1* appears to be conserved. In S. cerevisiae, Pet111 is located on the matrix side of the mitochondrial inner membrane, where it binds the 5′ untranslated region of *COX2* mRNA and facilitates its translation (70). The gene is highly diverged in other yeast species but has retained its orthologous function (71). This appears also to be the case with C. albicans as the corresponding mutant lacked cytochrome oxidase, although the mechanism of that loss was not examined. With regard to *AEP1*, functional conservation appears likely. C. albicans *aep1Δ*/*Δ* mutants failed to express mitochondrial ATP synthase. *ScAEP1* is required specifically for translation of *OLI1* mRNA, which encodes subunit 9 of ATP synthase (72). However, in addition to loss of ATP synthase, complex IV was also absent in C. albicans *aep1Δ*/*Δ* mutants and the contents of complexes I and III were reduced. It is not known if these broader changes occur in S. cerevisiae and whether they are secondary to loss of ATP synthase or reflect a more general role of Aep1 in synthesis and assembly of the ETC.

Phenotypic characterization of the respiratory mutants showed that any disruption of respiration had significant biological effects. The yeast-hypha transition is a critical virulence attribute of C. albicans and is influenced by respiration, but various studies have reached different conclusions regarding its role. Reduced respiration in hyphae, the stimulatory effect of ETC inhibitors, and increased filamentation under hypoxic conditions all suggest that a reduction in respiration stimulates the transition to the hyphal form (56, 58). Conversely, a positive role for respiration is suggested by studies showing that hypoxic conditions prevent filamentation (57, 61) and that phenazines and methylene blue, which short-circuit electron flow through the ETC, are inhibitory (59, 60). Genetic studies also support the idea of a positive role as mutants lacking *NDH51*, *GOA1*, or *COX4* are deficient in filamentation (8, 10, 16, 59). The results reported here showed that the morphological response of respiration-deficient mutants was largely independent of the particular block in respiration and had either no effect or a negative effect depending upon the inducing environment. This was most clearly illustrated by the almost complete lack of filamentation of the mutants on the surface of YPD-plus-serum medium versus formation of abundant hyphae when embedded in the medium. This may reflect differences between the environments with respect to energy metabolism or signal processing (59), but, regardless of the mechanism, the variability of responses in relationship to the environment might account for the disparity in the prior conclusions given the wide range of inducing conditions employed.

Fluconazole is a highly utilized antifungal, and efforts to enhance its efficacy and counter resistance are needed (73). Thus, it was of interest that all of the deletion mutants were hypersensitive to fluconazole irrespective of which respiratory complex was affected. The mechanistic underpinnings of this phenotype remain to be determined; however, the observations of Benhamou et al. (74) may be relevant. Those investigators demonstrated that azoles localize to the mitochondria (74) and that azole derivatives designed to direct localization to the endoplasmic reticulum (ER), where the target of azoles, Erg11, resides, show enhanced antifungal activity (75). If mitochondrial accumulation of azoles is dependent upon mitochondrial membrane potential, then respiratory mutants, because of a reduced mitochondrial membrane potential, may divert less fluconazole to mitochondria, allowing enhanced concentrations at the site of action, and this would be reflected in an enhanced sensitivity to azoles.

Reduced respiration limited growth under optimal conditions and might have a more substantial effect under stress conditions in the host niche. Available studies have shown that mitochondrial function in general (6, 7) and electron transport chain components specifically (8, 9, 11) are essential for C. albicans virulence in mice. This was mirrored in the G. mellonella infection model, where loss of any one component of the electron transport chain attenuated virulence. This suggests that respiration *per se* is a key requirement for virulence. The results further support the idea of the importance of the mitochondrion in the biology of C. albicans and its relevance as a potential therapeutic target (21).

## MATERIALS AND METHODS

### Strains and culture conditions.

C. albicans strains used are listed in Table S1 in the supplemental material. Strains were routinely cultured on YPD (76) or synthetic complete (SC) medium (United States Biological). Yeast extract-peptone-glucose (YPG) (76) was used to screen for respiration-deficient strains. Carbon source utilization was assessed on yeast nitrogen base (YNB) medium (76) containing 100 mM glucose, 100 mM pyruvate, 3% glycerol, 2% ethanol, or 2% sodium acetate. Media were solidified with 2% agar.

10.1128/mBio.00300-19.4 TABLE S1 Genotypes. Download Table S1, DOCX file, 0.03 MB. Copyright © 2019 Sun et al. 2019 Sun et al. This content is distributed under the terms of the Creative Commons Attribution 4.0 International license.

10.1128/mBio.00300-19.5 TABLE S2 Primers used for gene deletion, integration mapping, and complementation. Download Table S2, DOCX file, 0.03 MB. Copyright © 2019 Sun et al. 2019 Sun et al. This content is distributed under the terms of the Creative Commons Attribution 4.0 International license.

10.1128/mBio.00300-19.6 TABLE S3 Primers for plasmid pleuARGleu construction and verification of integration at *LEU2*. Download Table S3, DOCX file, 0.01 MB. Copyright © 2019 Sun et al. 2019 Sun et al. This content is distributed under the terms of the Creative Commons Attribution 4.0 International license.

10.1128/mBio.00300-19.7 TABLE S4 Primers used in restoration of the *ARG4* locus. Download Table S4, DOCX file, 0.01 MB. Copyright © 2019 Sun et al. 2019 Sun et al. This content is distributed under the terms of the Creative Commons Attribution 4.0 International license.

10.1128/mBio.00300-19.8 TABLE S5 Primers used in construction of GFP-tagged genes. Download Table S5, DOCX file, 0.02 MB. Copyright © 2019 Sun et al. 2019 Sun et al. This content is distributed under the terms of the Creative Commons Attribution 4.0 International license.

For growth rate determinations, the optical density at 595 nm (OD_595_) was monitored at 30°C in a Tecan plate reader. Growth rates were calculated in R (77) using the easy linear method implemented in the “growthrates” package (78) with the *h* value set to encompass approximately two cell doublings. Four to five independent determinations were made for each stain and medium.

### Identification of clade-specific mitochondrial proteins.

A BLASTP (79) comparison of the predicted proteins from assembly 21 of the C. albicans genome (http://www.candidagenome.org/) with those of S. cerevisiae (http://downloads.yeastgenome.org/sequence/S288C_reference/orf_protein/) and Aspergillus nidulans (http://www.aspergillusgenome.org/) identified a subset of 1,349 nonconserved or poorly conserved C. albicans proteins with an E value of >10^−6^. InParanoid (80) and reciprocal BLAST comparison of this subset against the genomes of Candida tropicalis, Candida guilliermondii, Candida lusitaniae, and Lodderomyces elongisporus (https://www.broadinstitute.org/fungal-genome-initiative/comparative-candida-genomic-project) or of C. parapsilosis (http://www.sanger.ac.uk/resources/downloads/fungi/) and Debaryomyces hansenii (http://genome.jgi.doe.gov/) identified 419 orthologous protein families present in all eight species. Putative mitochondrial proteins among the orthologous groups were identified using MitoPred (36), TargetP (37), and Predotar (38). Among 86 of the orthologous groups, mitochondrial localization was indicated for five or more family members by at least one algorithm. These were further analyzed by PsiBLAST (81) comparison of the C. albicans ortholog with the nonredundant database at NCBI to detect more distantly related non-CTG clade orthologs that may have been missed in the initial, less sensitive BLAST screen. Proteins for which the second iteration identified potential orthologs in non-CTG clade species (E value <10^−4^) were removed, leaving 25 putative clade-specific mitochondrial proteins.

### Strain constructions.

Deletion mutants were constructed in C. albicans strain SN152 essentially as described by Noble and Johnson (82). Details are provided in Text S1 in the supplemental material.

10.1128/mBio.00300-19.1 TEXT S1 Supplemental Methods. Download Text S1, DOCX file, 0.02 MB. Copyright © 2019 Sun et al. 2019 Sun et al. This content is distributed under the terms of the Creative Commons Attribution 4.0 International license.

### Primers.

The primers used in this study are listed in Table S2, Table S3, Table S4, and Table S5.

### Respiration measurements.

Preliminary experiments showed maximal respiration in cells in early log phase, as previously reported (83). Strains that had been cultured to the stationary phase in YPD broth were used to inoculate fresh YPD medium (2.5 × 10^6^ cells/ml), and cultures were incubated 4 h at 30°C with vigorous aeration. Aliquots of culture were mixed with prewarmed YPD, and O_2_ utilization was measured using a Hansatech Oxygraph system (Hansatech Instruments). Utilization rates were normalized to the OD_595_ of the sample. Contributions of ETC and alternative oxidase were assessed by addition of KCN to inhibit cytochrome oxidase or of salicylhydroxamic acid (SHAM) to inhibit alternative oxidase (42). KCN (100 mM)–Tris (pH 8) was added to reach a final concentration of 1 mM. SHAM (500 mM)–dimethyl sulfoxide (DMSO) was added to reach a final concentration of 5 mM. DMSO alone had no effect. Rotenone (25 μM) was added to assess the contribution of rotenone-insensitive alternative NADH-Q oxidoreductase (45). At least three independent determinations were made for each strain.

### Isolation of mitochondria.

Mitochondria were isolated by the use of a modification of previously described procedures (84). Details of the protocol are described in Text S1.

### Blue native gel electrophoresis.

Purified mitochondria were solubilized with 1% n-dodecylmaltoside (85) and electrophoresed on 5% to 15% polyacrylamide gradient gels as described previously (46) except that 0.02% n-dodecylmaltoside was added to the cathode buffer and 0.02% Coomassie G250 was continuously present in the cathode buffer. Gels were destained in 10% acetic acid–100 mM ammonium acetate–50% methanol (86) and stained with Imperial protein stain (Pierce). In-gel enzyme activity assays were performed as described previously (87).

### Cell fractionation and SDS-PAGE of GFP-tagged strains.

Total cell lysates and gradient-purified mitochondria were prepared from GFP-tagged strains. Total lysates were centrifuged 15 min at 13,000 × *g* to prepare mitochondrion-free lysates. Samples (100 μg protein) were separated on 10% acrylamide–0.1% SDS gels according to Laemmli (88) except that, to prevent denaturation of GFP, samples were not boiled (52). Gels were equilibrated in 100 mM Tris (pH 8.1) twice for 10 min each time, incubated for 5 min in 50 mM Tris (pH 8.1)–100 mM sodium dithionate, and imaged using a GE ImageQuant LAS-4010 imaging system. The use of sodium dithionate reduced flavin-containing proteins and decreased their fluorescence (89). For relative quantitation, mitochondrial fractions containing 10 μg protein (*NUO3*-, *NUO4*-, and *COX8*-tagged strains) or 50 μg protein (all others) were subjected to electrophoresis on the same gel and imaged. Quantitative analysis of the images was performed with ImageQuant TL ver. 8.1. Mitochondrial extracts were also fractionated by blue native gel electrophoresis and imaged for fluorescence.

### Confocal microscopy.

GFP-tagged strains were cultured to the stationary phase in YPD at 30°C and inoculated into fresh YPD at a density of approximately 1 × 10^6^ cells/ml. After 3 to 4 h of incubation, cells were labeled with MitoTracker Red CMXRos (Molecular Probes), fixed with formaldehyde, and imaged with a Zeiss LSM510 Meta confocal laser scanning microscope equipped with a Plan-Apochromat 100×/1.4 oil objective. Details of the protocol are provided in Text S1.

Colocalization was analyzed as described by Costes et al. (51) using the Coloc 2 algorithm as implemented in the Fiji package of ImageJ (90). Images were processed with a 2-pixel median filter, and background was subtracted with a 1-pixel sliding paraboloid before quantitation. A minimum of 30 cells from at least two independent samples were analyzed, and the Costes significance test was based on 100 randomizations.

### Germ tube and induction of hyphae.

Germ tube formation was assessed in medium 199–100 mM morpholinepropanesulfonic acid (MOPS; pH 7.5) inoculated to reach a density of approximately 2.5 × 10^6^ cells/ml with cells cultured 48 h in SC medium. To assess filamentation on agar surfaces, stationary-phase cells were diluted to 5 × 10^7^ cells/ml and 2 μl of suspension was spotted on medium 199 (Gibco)–100 mM MOPS (pH 7.5) or YPD plus 10% fetal calf serum or YNBAGN (59) and incubated at 37°C. The effect of embedded conditions was tested by suspending approximately 50 cells in 25 ml YPD-plus-10% serum agar (held at 47°C) followed by pouring the reaction mixture into petri plates. After solidification, the plates were incubated at 37°C.

### Fluconazole sensitivity.

Strains were cultured to the stationary phase in SC medium. Ten-fold serial dilutions with densities ranging from 1 × 10^8^ to 1 × 10^5^ cells/ml were prepared, and 2 μl of each was spotted on YPD or YPD with fluconazole (8 μg/ml). Plates were incubated at 30°C.

### G. mellonella survival assay.

The survival assay was conducted essentially as described by Fuchs et al. (62, 91) using groups of 10 larvae (Vanderhorst, Inc.) (330 ± 20 mg) injected with 5 × 10^6^ cells in 10 μl PBS or PBS alone. Larvae were incubated at 37°C, and the number of dead larvae was scored daily. Kaplan-Meier analysis was performed, log rank statistics were calculated in R (77) using the package “survival” (92), and survival curves were plotted using “survminer” (93).
