# Supplementary figures and images for: Unique, Diverged, and Conserved Mitochondrial Functions Influencing Candida albicans Respiration
Source: mBio. 2019 Jun 25;10(3):e00300-19. doi: 10.1128/mBio.00300-19 (PMC6593398; doi:10.1128/mBio.00300-19)

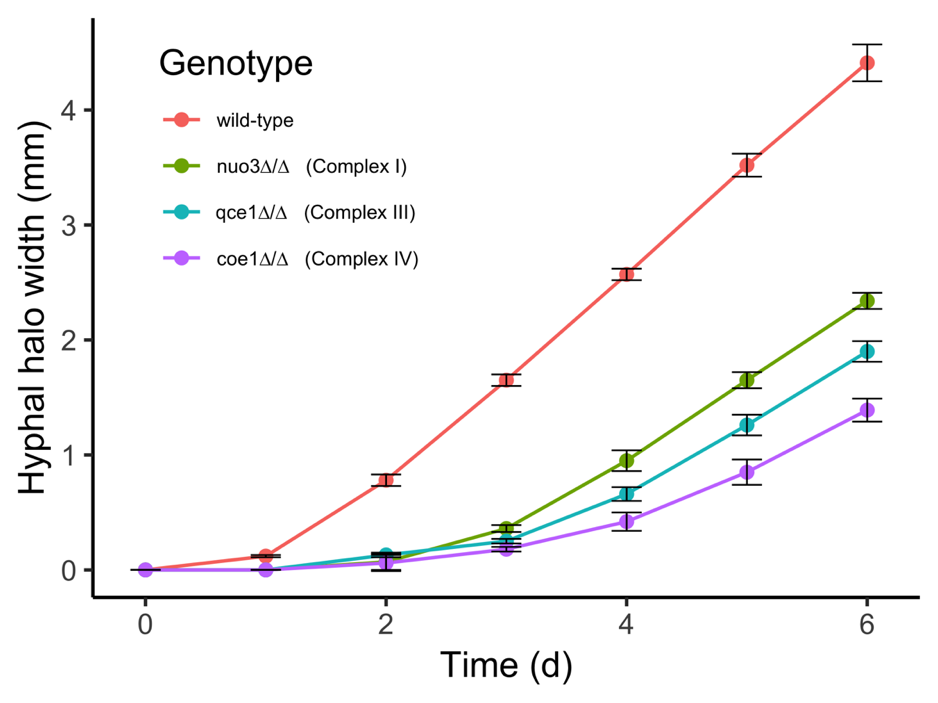

Supplement: FIG S1 [file mBio.00300-19-sf001.tif]

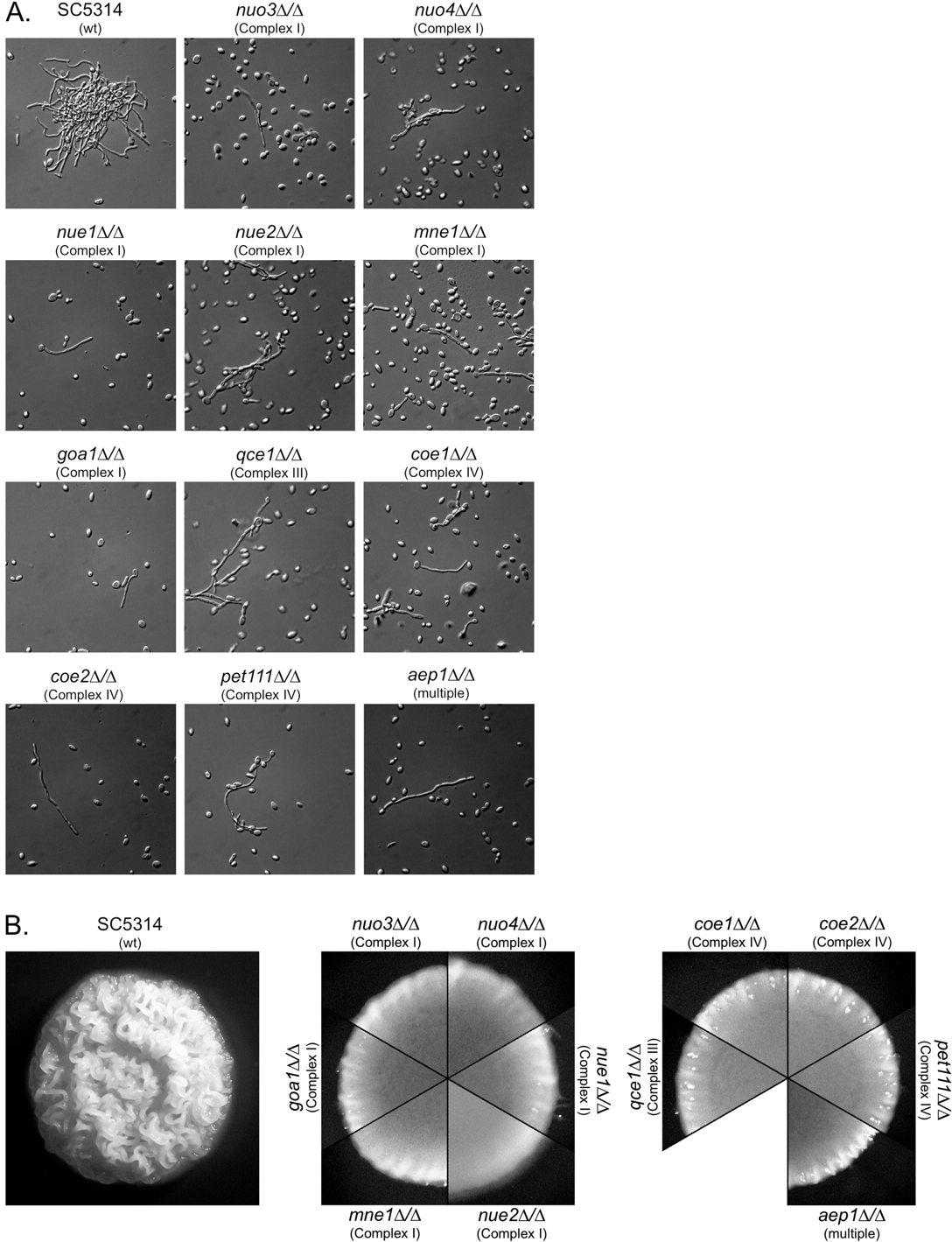

Supplement: FIG S2 [file mBio.00300-19-sf002.tif]
