# Supplementary material for: Unique, Diverged, and Conserved Mitochondrial Functions Influencing Candida albicans Respiration
Source: mBio. 2019 Jun 25;10(3):e00300-19. doi: 10.1128/mBio.00300-19 (PMC6593398; doi:10.1128/mBio.00300-19)
Supplement: TABLE S1 [file mBio.00300-19-st001.docx]

**Table S1**

| **Strain** | **Genotype** | **Reference** |
| --- | --- | --- |
| SC5314 | wild-type | (1) |
| SN152 | *his1*∆/*his1*∆ *leu2*∆ /*leu2*∆ *arg4*∆/*arg4*∆  *URA3*/*ura3*∆::*imm^434^* *IRO1*/*iro1*∆::*imm^434^* | (2) |
| SN250 | *his1*∆/*his1*∆ *leu2*∆::*CdHIS1* /*leu2*∆::*CmLEU2* *arg4*∆/*arg4*∆  *URA3*/*ura3*∆::*imm^434^* *IRO1*/*iro1*∆::*imm^434^* | (3) |
| *GOA1*ms-11 | *GOA1*/*goa1*∆::Cm*LEU2* *leu2*∆/*leu2*∆ *his1*∆/*his1*∆ *arg4*∆/*arg4*∆  *URA3*/*ura3*∆::*imm^434^* *IRO1*/*iro1*∆::*imm^434^* | This work |
| *GOA1*ms-25 | *goa1*∆::Cm*LEU2*/*goa1*∆::Cd*HIS1* *leu2*∆/*leu2*∆ *his1*∆/*his1*∆ *arg4*∆/*arg4*∆  *URA3*/*ura3*∆::*imm^434^* *IRO1*/*iro1*∆::*imm^434^* | This work |
| *GOA1*ms-25-31 | *goa1*∆::Cm*LEU2*/*goa1*∆::Cd*HIS1* *leu2*∆::*ARG4*-*GOA1*/*leu2*∆ *his1*∆/*his1*∆ *arg4*∆/*arg4*∆  *URA3*/*ura3*∆::*imm^434^* *IRO1*/*iro1*∆::*imm^434^* | This work |
| *GOA1*ms-41 | *goa1*∆::Cm*LEU2*/*goa1*∆::Cd*HIS1* *leu2*∆/*leu2*∆ *his1*∆/*his1*∆ *ARG4*/*arg4*∆  *URA3*/*ura3*∆::*imm^434^* *IRO1*/*iro1*∆::*imm^434^* | This work |
| mt94-13 | ORF19.94/orf19.94∆::Cm*LEU2* *leu2*∆/*leu2*∆ *his1*∆/*his1*∆ *arg4*∆/*arg4*∆  *URA3*/*ura3*∆::*imm^434^* *IRO1*/*iro1*∆::*imm^434^* | This work |
| mt94-21 | orf19.94∆::Cm*LEU2*/orf19.94∆::Cd*HIS1* *leu2*∆/*leu2*∆ *his1*∆/*his1*∆ *arg4*∆/*arg4*∆  *URA3*/*ura3*∆::*imm^434^* *IRO1*/*iro1*∆::*imm^434^* | This work |
| mt102-11 | *AEP1*/*aep1*∆::Cm*LEU2* *leu2*∆/*leu2*∆ *his1*∆/*his1*∆ *arg4*∆/*arg4*∆  *URA3*/*ura3*∆::*imm^434^* *IRO1*/*iro1*∆::*imm^434^* | This work |
| mt102-22 | *aep1*∆::Cm*LEU2*/*aep1*∆::Cd*HIS1* *leu2*∆/*leu2*∆ *his1*∆/*his1*∆ *arg4*∆/*arg4*∆  *URA3*/*ura3*∆::*imm^434^* *IRO1*/*iro1*∆::*imm^434^* | This work |
| mt102-34 | *aep1*∆::Cm*LEU2*/*aep1*∆::Cd*HIS1* *leu2*∆::*ARG4*-*AEP1*/*leu2*∆ *his1*∆/*his1*∆ *arg4*∆/*arg4*∆  *URA3*/*ura3*∆::*imm^434^* *IRO1*/*iro1*∆::*imm^434^* | This work |
| mt102-41 | *aep1*∆::Cm*LEU2*/*aep1*∆::Cd*HIS1* *leu2*∆/*leu2*∆ *his1*∆/*his1*∆ *ARG4*/*arg4*∆  *URA3*/*ura3*∆::*imm^434^* *IRO1*/*iro1*∆::*imm^434^* | This work |
| mt230-11 | *PET111*/*pet111*∆::Cm*LEU2* *leu2*∆/*leu2*∆ *his1*∆/*his1*∆ *arg4*∆/*arg4*∆  *URA3*/*ura3*∆::*imm^434^* *IRO1*/*iro1*∆::*imm^434^* | This work |
| mt230-21 | *pet111*∆::Cm*LEU2*/*pet111*∆::Cd*HIS1* *leu2*∆/*leu2*∆ *his1*∆/*his1*∆ *arg4*∆/*arg4*∆  *URA3*/*ura3*∆::*imm^434^* *IRO1*/*iro1*∆::*imm^434^* | This work |
| mt230-39 | *pet111*∆::Cm*LEU2*/*pet111*∆::Cd*HIS1* *leu2*∆::*ARG4*-*PET111*/*leu2*∆ *his1*∆/*his1*∆ *arg4*∆/*arg4*∆  *URA3*/*ura3*∆::*imm^434^* *IRO1*/*iro1*∆::*imm^434^* | This work |
| mt230-41 | *pet111*∆::Cm*LEU2*/*pet111*∆::Cd*HIS1* *leu2*∆/*leu2*∆ *his1*∆/*his1*∆ *ARG4*/*arg4*∆  *URA3*/*ura3*∆::*imm^434^* *IRO1*/*iro1*∆::*imm^434^* | This work |
| mt265-13 | ORF19.265/orf19.265∆::Cm*LEU2* *leu2*∆/*leu2*∆ *his1*∆/*his1*∆ *arg4*∆/*arg4*∆  *URA3*/*ura3*∆::*imm^434^* *IRO1*/*iro1*∆::*imm^434^* | This work |
| mt265-23 | orf19.265∆::Cm*LEU2*/orf19.265∆::Cd*HIS1* *leu2*∆/*leu2*∆ *his1*∆/*his1*∆ *arg4*∆/*arg4*∆  *URA3*/*ura3*∆::*imm^434^* *IRO1*/*iro1*∆::*imm^434^* | This work |
| mt411-12 | ORF19.411/orf19.411∆::Cm*LEU2* *leu2*∆/*leu2*∆ *his1*∆/*his1*∆ *arg4*∆/*arg4*∆  *URA3*/*ura3*∆::*imm^434^* *IRO1*/*iro1*∆::*imm^434^* | This work |
| mt411-22 | orf19.411∆::Cm*LEU2*/orf19.411∆::Cd*HIS1* *leu2*∆/*leu2*∆ *his1*∆/*his1*∆ *arg4*∆/*arg4*∆  *URA3*/*ura3*∆::*imm^434^* *IRO1*/*iro1*∆::*imm^434^* | This work |
| mt527-11 | ORF19.265/orf19.527∆::Cm*LEU2* *leu2*∆/*leu2*∆ *his1*∆/*his1*∆ *arg4*∆/*arg4*∆  *URA3*/*ura3*∆::*imm^434^* *IRO1*/*iro1*∆::*imm^434^* | This work |
| mt679-11 | *FGR39*/*fgr39*∆::Cm*LEU2* *leu2*∆/*leu2*∆ *his1*∆/*his1*∆ *arg4*∆/*arg4*∆  *URA3*/*ura3*∆::*imm^434^* *IRO1*/*iro1*∆::*imm^434^* | This work |
| mt679-21 | *fgr39*∆::Cm*LEU2*/*fgr39*∆::Cd*HIS1* *leu2*∆/*leu2*∆ *his1*∆/*his1*∆ *arg4*∆/*arg4*∆  *URA3*/*ura3*∆::*imm^434^* *IRO1*/*iro1*∆::*imm^434^* | This work |
| mt935-15 | *AGA1*/*aga1*∆::Cm*LEU2* *leu2*∆/*leu2*∆ *his1*∆/*his1*∆ *arg4*∆/*arg4*∆  *URA3*/*ura3*∆::*imm^434^* *IRO1*/*iro1*∆::*imm^434^* | This work |
| mt935-21 | *aga1*∆::Cm*LEU2*/*aga1*∆::Cd*HIS1* *leu2*∆/*leu2*∆ *his1*∆/*his1*∆ *arg4*∆/*arg4*∆  *URA3*/*ura3*∆::*imm^434^* *IRO1*/*iro1*∆::*imm^434^* | This work |
| mt1179-11 | *NUO3*/*nuo3*∆::Cm*LEU2* *leu2*∆/*leu2*∆ *his1*∆/*his1*∆ *arg4*∆/*arg4*∆  *URA3*/*ura3*∆::*imm^434^* *IRO1*/*iro1*∆::*imm^434^* | This work |
| mt1179-21 | *nuo3*∆::Cm*LEU2*/*nuo3*∆::Cd*HIS1* *leu2*∆/*leu2*∆ *his1*∆/*his1*∆ *arg4*∆/*arg4*∆  *URA3*/*ura3*∆::*imm^434^* *IRO1*/*iro1*∆::*imm^434^* | This work |
| mt1179-31 | *nuo3*∆::Cm*LEU2*/nuo3∆::Cd*HIS1* *leu2*∆::*ARG4*-*NUO3*/*leu2*∆ *his1*∆/*his1*∆ *arg4*∆/*arg4*∆  *URA3*/*ura3*∆::*imm^434^* *IRO1*/*iro1*∆::*imm^434^* | This work |
| mt1179-41 | *nuo3*∆::Cm*LEU2*/nuo3∆::Cd*HIS1* *leu2*∆/*leu2*∆ *his1*∆/*his1*∆ *ARG4*/*arg4*∆  *URA3*/*ura3*∆::*imm^434^* *IRO1*/*iro1*∆::*imm^434^* | This work |
| mt1287-12 | ORF19.1287/orf19.1287∆::Cm*LEU2* *leu2*∆/*leu2*∆ *his1*∆/*his1*∆ *arg4*∆/*arg4*∆  *URA3*/*ura3*∆::*imm^434^* *IRO1*/*iro1*∆::*imm^434^* | This work |
| mt1287-21 | orf19.1287∆::Cm*LEU2*/orf19.1287∆::Cd*HIS1* *leu2*∆/*leu2*∆ *his1*∆/*his1*∆ *arg4*∆/*arg4*∆  *URA3*/*ura3*∆::*imm^434^* *IRO1*/*iro1*∆::*imm^434^* | This work |
| mt1344-11 | ORF19.1344/orf19.1344∆::Cm*LEU2* *leu2*∆/*leu2*∆ *his1*∆/*his1*∆ *arg4*∆/*arg4*∆  *URA3*/*ura3*∆::*imm^434^* *IRO1*/*iro1*∆::*imm^434^* | This work |
| mt1344-23 | orf19.1344∆::Cm*LEU2*/orf19.1344∆::Cd*HIS1* *leu2*∆/*leu2*∆ *his1*∆/*his1*∆ *arg4*∆/*arg4*∆  *URA3*/*ura3*∆::*imm^434^* *IRO1*/*iro1*∆::*imm^434^* | This work |
| mt1371-11 | *COE1*/*coe1*∆::Cm*LEU2* *leu2*∆/*leu2*∆ *his1*∆/*his1*∆ *arg4*∆/*arg4*∆  *URA3*/*ura3*∆::*imm^434^* *IRO1*/*iro1*∆::*imm^434^* | This work |
| mt1371-21 | *coe1*∆::Cm*LEU2*/*coe1*∆::Cd*HIS1* *leu2*∆/*leu2*∆ *his1*∆/*his1*∆ *arg4*∆/*arg4*∆  *URA3*/*ura3*∆::*imm^434^* *IRO1*/*iro1*∆::*imm^434^* | This work |
| mt1371-31 | *coe1*∆::Cm*LEU2*/*coe1*∆::Cd*HIS1* *leu2*∆::*ARG4*-*COE1*/*leu2*∆ *his1*∆/*his1*∆ *arg4*∆/*arg4*∆  *URA3*/*ura3*∆::*imm^434^* *IRO1*/*iro1*∆::*imm^434^* | This work |
| mt1371-41 | *coe1*∆::Cm*LEU2*/*coe1*∆::Cd*HIS1* *leu2*∆/*leu2*∆ *his1*∆/*his1*∆ *ARG4*/*arg4*∆  *URA3*/*ura3*∆::*imm^434^* *IRO1*/*iro1*∆::*imm^434^* | This work |
| mt1748-11 | ORF19.1748/orf19.1748∆::Cm*LEU2* *leu2*∆/*leu2*∆ *his1*∆/*his1*∆ *arg4*∆/*arg4*∆  *URA3*/*ura3*∆::*imm^434^* *IRO1*/*iro1*∆::*imm^434^* | This work |
| mt1748-21 | orf19.1784∆::Cm*LEU2*/orf19.1784∆::Cd*HIS1* *leu2*∆/*leu2*∆ *his1*∆/*his1*∆ *arg4*∆/*arg4*∆  *URA3*/*ura3*∆::*imm^434^* *IRO1*/*iro1*∆::*imm^434^* | This work |
| mt1873-11 | ORF19.1873/orf19.1873∆::Cm*LEU2* *leu2*∆/*leu2*∆ *his1*∆/*his1*∆ *arg4*∆/*arg4*∆  *URA3*/*ura3*∆::*imm^434^* *IRO1*/*iro1*∆::*imm^434^* | This work |
| mt1873-21 | orf19.1873∆::Cm*LEU2*/orf19.1873∆::Cd*HIS1* *leu2*∆/*leu2*∆ *his1*∆/*his1*∆ *arg4*∆/*arg4*∆  *URA3*/*ura3*∆::*imm^434^* *IRO1*/*iro1*∆::*imm^434^* | This work |
| mt2513-11 | *MNE1*/*mne1*∆::Cm*LEU2* *leu2*∆/*leu2*∆ *his1*∆/*his1*∆ *arg4*∆/*arg4*∆  *URA3*/*ura3*∆::*imm^434^* *IRO1*/*iro1*∆::*imm^434^* | This work |
| mt2513-21 | *mne1*∆::Cm*LEU2*/*mne1*∆::Cd*HIS1* *leu2*∆/*leu2*∆ *his1*∆/*his1*∆ *arg4*∆/*arg4*∆  *URA3*/*ura3*∆::*imm^434^* *IRO1*/*iro1*∆::*imm^434^* | This work |
| mt2513-32 | *mne1*∆::Cm*LEU2*/*mne1*∆::Cd*HIS1* *leu2*∆::*ARG4*-*MNE1*/*leu2*∆ *his1*∆/*his1*∆ *arg4*∆/*arg4*∆  *URA3*/*ura3*∆::*imm^434^* *IRO1*/*iro1*∆::*imm^434^* | This work |
| mt2513-41 | *mne1*∆::Cm*LEU2*/*mne1*∆::Cd*HIS1* *ARG4*/*arg4*∆  *leu2*∆/*leu2*∆ *his1*∆/*his1*∆ *URA3*/*ura3*∆::*imm^434^* *IRO1*/*iro1*∆::*imm^434^* | This work |
| mt2650-11 | ORF19.2650/orf19.2650∆::Cm*LEU2* *leu2*∆/*leu2*∆ *his1*∆/*his1*∆ *arg4*∆/*arg4*∆  *URA3*/*ura3*∆::*imm^434^* *IRO1*/*iro1*∆::*imm^434^* | This work |
| mt2650-23 | orf19.2650∆::Cm*LEU2*/orf19.2650∆::Cd*HIS1* *leu2*∆/*leu2*∆ *his1*∆/*his1*∆ *arg4*∆/*arg4*∆ *URA3*/*ura3*∆::*imm^434^* *IRO1*/*iro1*∆::*imm^434^* | This work |
| mt2819-15 | *NUE1*/*nue1*∆::Cm*LEU2* *leu2*∆/*leu2*∆ *his1*∆/*his1*∆ *arg4*∆/*arg4*∆  *URA3*/*ura3*∆::*imm^434^* *IRO1*/*iro1*∆::*imm^434^* | This work |
| mt2819-21 | *nue1*∆::Cm*LEU2*/*nue1*∆::Cd*HIS1* *leu2*∆/*leu2*∆ *his1*∆/*his1*∆ *arg4*∆/*arg4*∆  *URA3*/*ura3*∆::*imm^434^* *IRO1*/*iro1*∆::*imm^434^* | This work |
| mt2819-33 | *nue1*∆::Cm*LEU2*/*nue1*∆::Cd*HIS1* *leu2*∆::*ARG4*-*NUE1*/*leu2*∆ *his1*∆/*his1*∆ *arg4*∆/*arg4*∆  *URA3*/*ura3*∆::*imm^434^* *IRO1*/*iro1*∆::*imm^434^* | This work |
| mt2819-41 | *nue1*∆::Cm*LEU2*/*nue1*∆::Cd*HIS1* *leu2*∆/*leu2*∆ *his1*∆/*his1*∆ *ARG4*/*arg4*∆  *URA3*/*ura3*∆::*imm^434^* *IRO1*/*iro1*∆::*imm^434^* | This work |
| mt3563-11 | ORF19.3563/orf19.3563∆::Cm*LEU2* *leu2*∆/*leu2*∆ *his1*∆/*his1*∆ *arg4*∆/*arg4*∆  *URA3*/*ura3*∆::*imm^434^* *IRO1*/*iro1*∆::*imm^434^* | This work |
| mt3563-24 | orf19.3563∆::Cm*LEU2*/orf19.3563∆::Cd*HIS1* *leu2*∆/*leu2*∆ *his1*∆/*his1*∆ *arg4*∆/*arg4*∆  *URA3*/*ura3*∆::*imm^434^* *IRO1*/*iro1*∆::*imm^434^* | This work |
| mt4467-11 | *NUE2*/*nue2*∆::Cm*LEU2* *leu2*∆/*leu2*∆ *his1*∆/*his1*∆ *arg4*∆/*arg4*∆  *URA3*/*ura3*∆::*imm^434^* *IRO1*/*iro1*∆::*imm^434^* | This work |
| mt4467-25 | *nue2*∆::Cm*LEU2*/*nue2*∆::Cd*HIS1* *leu2*∆/*leu2*∆ *his1*∆/*his1*∆ *arg4*∆/*arg4*∆  *URA3*/*ura3*∆::*imm^434^* *IRO1*/*iro1*∆::*imm^434^* | This work |
| mt4467-31 | *nue2*∆::Cm*LEU2*/*nue2*∆::Cd*HIS1* *leu2*∆::*ARG4*-*NUE2*/*leu2*∆ *his1*∆/*his1*∆ *arg4*∆/*arg4*∆  *URA3*/*ura3*∆::*imm^434^* *IRO1*/*iro1*∆::*imm^434^* | This work |
| mt4467-41 | *nue2*∆::Cm*LEU2*/*nue2*∆::Cd*HIS1* *leu2*∆/*leu2*∆ *his1*∆/*his1*∆ *ARG4*/*arg4*∆  *URA3*/*ura3*∆::*imm^434^* *IRO1*/*iro1*∆::*imm^434^* | This work |
| mt4553-11 | ORF19.4553/orf19.4553∆::Cm*LEU2* *leu2*∆/*leu2*∆ *his1*∆/*his1*∆ *arg4*∆/*arg4*∆  *URA3*/*ura3*∆::*imm^434^* *IRO1*/*iro1*∆::*imm^434^* | This work |
| mt4553-21 | orf19.4553∆::Cm*LEU2*/orf19.4553∆::Cd*HIS1* *leu2*∆/*leu2*∆ *his1*∆/*his1*∆ *arg4*∆/*arg4*∆  *URA3*/*ura3*∆::*imm^434^* *IRO1*/*iro1*∆::*imm^434^* | This work |
| mt4734-11 | ORF19.4734/orf19.4734∆::Cm*LEU2* *leu2*∆/*leu2*∆ *his1*∆/*his1*∆ *arg4*∆/*arg4*∆  *URA3*/*ura3*∆::*imm^434^* *IRO1*/*iro1*∆::*imm^434^* | This work |
| mt4734-21 | orf19.4734∆::Cm*LEU2*/orf19.4734∆::Cd*HIS1* *leu2*∆/*leu2*∆ *his1*∆/*his1*∆ *arg4*∆/*arg4*∆  *URA3*/*ura3*∆::*imm^434^* *IRO1*/*iro1*∆::*imm^434^* | This work |
| mt4795-11 | ORF19.4795/orf19.4795∆::Cm*LEU2* *leu2*∆/*leu2*∆ *his1*∆/*his1*∆ *arg4*∆/*arg4*∆  *URA3*/*ura3*∆::*imm^434^* *IRO1*/*iro1*∆::*imm^434^* | This work |
| mt4795-21 | orf19.4795∆::Cm*LEU2*/orf19.4795∆::Cd*HIS1* *leu2*∆/*leu2*∆ *his1*∆/*his1*∆ *arg4*∆/*arg4*∆  *URA3*/*ura3*∆::*imm^434^* *IRO1*/*iro1*∆::*imm^434^* | This work |
| mt4895-12 | ORF19.4895/orf19.4895∆::Cm*LEU2* *leu2*∆/*leu2*∆ *his1*∆/*his1*∆ *arg4*∆/*arg4*∆  *URA3*/*ura3*∆::*imm^434^* *IRO1*/*iro1*∆::*imm^434^* | This work |
| mt4895-23 | orf19.4895∆::Cm*LEU2*/orf19.4895∆::Cd*HIS1* *leu2*∆/*leu2*∆ *his1*∆/*his1*∆ *arg4*∆/*arg4*∆  *URA3*/*ura3*∆::*imm^434^* *IRO1*/*iro1*∆::*imm^434^* | This work |
| mt5077-11 | *NUO4/nuo4*∆::Cm*LEU2* *leu2*∆/*leu2*∆ *his1*∆/*his1*∆ *arg4*∆/*arg4*∆  *URA3*/*ura3*∆::*imm^434^* *IRO1*/*iro1*∆::*imm^434^* | This work |
| mt5077-21 | *nuo4*∆::Cm*LEU2*/*nuo4*∆::Cd*HIS1* *leu2*∆/*leu2*∆ *his1*∆/*his1*∆ *arg4*∆/*arg4*∆  *URA3*/*ura3*∆::*imm^434^* *IRO1*/*iro1*∆::*imm^434^* | This work |
| mt5077-31 | *nuo4*∆::Cm*LEU2*/*nuo4*∆::Cd*HIS1* *leu2*∆::*ARG4*-*NUO4*/*leu2*∆ *his1*∆/*his1*∆ *arg4*∆/*arg4*∆  *URA3*/*ura3*∆::*imm^434^* *IRO1*/*iro1*∆::*imm^434^* | This work |
| mt5077-41 | *nuo4*∆::Cm*LEU2*/*nuo4*∆::Cd*HIS1* *leu2*∆/*leu2*∆ *his1*∆/*his1*∆ *ARG4*/*arg4*∆  *URA3*/*ura3*∆::*imm^434^* *IRO1*/*iro1*∆::*imm^434^* | This work |
| mt5607-11 | ORF19.5607/orf19.5607∆::Cm*LEU2* *leu2*∆/*leu2*∆ *his1*∆/*his1*∆ *arg4*∆/*arg4*∆  *URA3*/*ura3*∆::*imm^434^* *IRO1*/*iro1*∆::*imm^434^* | This work |
| mt5607-25 | orf19.5607∆::Cm*LEU2*/orf19.5607∆::Cd*HIS1* *leu2*∆/*leu2*∆ *his1*∆/*his1*∆ *arg4*∆/*arg4*∆  *URA3*/*ura3*∆::*imm^434^* *IRO1*/*iro1*∆::*imm^434^* | This work |
| mt5607-25-31 | orf19.5607∆::Cm*LEU2*/orf19.5607∆::Cd*HIS1* *leu2*∆::*ARG4*-ORF19.5607/*leu2*∆ *his1*∆/*his1*∆ *arg4*∆/*arg4*∆  *URA3*/*ura3*∆::*imm^434^* *IRO1*/*iro1*∆::*imm^434^* | This work |
| mt5607-41 | orf19.5607∆::Cm*LEU2*/orf19.5607∆::Cd*HIS1* *leu2*∆/*leu2*∆ *his1*∆/*his1*∆ *ARG4*/*arg4*∆  *URA3*/*ura3*∆::*imm^434^* *IRO1*/*iro1*∆::*imm^434^* | This work |
| mt6566-11 | *COE2*/*coe2*∆::Cm*LEU2* *leu2*∆/*leu2*∆ *his1*∆/*his1*∆ *arg4*∆/*arg4*∆  *URA3*/*ura3*∆::*imm^434^* *IRO1*/*iro1*∆::*imm^434^* | This work |
| mt6566-21 | *coe2*∆::Cm*LEU2*/*coe2*∆::Cd*HIS1* *leu2*∆/*leu2*∆ *his1*∆/*his1*∆ *arg4*∆/*arg4*∆  *URA3*/*ura3*∆::*imm^434^* *IRO1*/*iro1*∆::*imm^434^* | This work |
| mt6566-39 | *coe2*∆::Cm*LEU2*/*coe2*∆::Cd*HIS1* *leu2*∆::*ARG4*-*COE2*/*leu2*∆ *his1*∆/*his1*∆ *arg4*∆/*arg4*∆  *URA3*/*ura3*∆::*imm^434^* *IRO1*/*iro1*∆::*imm^434^* | This work |
| mt6566-41 | *coe2*∆::Cm*LEU2*/coe2∆::Cd*HIS1* *leu2*∆/*leu2*∆ *his1*∆/*his1*∆ *ARG4*/*arg4*∆  *URA3*/*ura3*∆::*imm^434^* *IRO1*/*iro1*∆::*imm^434^* | This work |
| mt6853-11 | ORF19.6853/orf19.6853∆::Cm*LEU2* *leu2*∆/*leu2*∆ *his1*∆/*his1*∆ *arg4*∆/*arg4*∆  *URA3*/*ura3*∆::*imm^434^* *IRO1*/*iro1*∆::*imm^434^* | This work |
| mt6853-21 | orf19.6853∆::Cm*LEU2*/orf19.6853∆::Cd*HIS1* *leu2*∆/*leu2*∆ *his1*∆/*his1*∆ *arg4*∆/*arg4*∆  *URA3*/*ura3*∆::*imm^434^* *IRO1*/*iro1*∆::*imm^434^* | This work |
| mt6918-11 | *QCE1*/*qce1*∆::Cm*LEU2* *leu2*∆/*leu2*∆ *his1*∆/*his1*∆ *arg4*∆/*arg4*∆  *URA3*/*ura3*∆::*imm^434^* *IRO1*/*iro1*∆::*imm^434^* | This work |
| mt6918-24 | *qce1*∆::Cm*LEU2*/*qce1*∆::Cd*HIS1* *leu2*∆/*leu2*∆ *his1*∆/*his1*∆ *arg4*∆/*arg4*∆  *URA3*/*ura3*∆::*imm^434^* *IRO1*/*iro1*∆::*imm^434^* | This work |
| mt6918-31 | *qce1*∆::Cm*LEU2*/*qce1*∆::Cd*HIS1* *leu2*∆::*ARG4*-*QCE1*/*leu2*∆ *his1*∆/*his1*∆ *arg4*∆/*arg4*∆  *URA3*/*ura3*∆::*imm^434^* *IRO1*/*iro1*∆::*imm^434^* | This work |
| mt6918-41 | *qce1*∆::Cm*LEU2*/*qce1*∆::Cd*HIS1* *leu2*∆/*leu2*∆ *his1*∆/*his1*∆ *ARG4*/*arg4*∆  *URA3*/*ura3*∆::*imm^434^* *IRO1*/*iro1*∆::*imm^434^* | This work |
| mt*COX*8-GFPγ | *COX*8::GFPγ-*ARG4*/*COX*8 *leu2*∆/*leu2*∆ *his1*∆/*his1*∆ *arg4*∆/*arg4*∆  *URA3*/*ura3*∆::*imm^434^* *IRO1*/*iro1*∆::*imm^434^* | This work |
| *GOA1*msGFPγ-11 | *GOA1*::GFPγ-*ARG4*/*goa1*::Cm*LEU2* *leu2*∆/*leu2*∆ *his1*∆/*his1*∆ *arg4*∆/*arg4*∆  *URA3*/*ura3*∆::*imm^434^* *IRO1*/*iro1*∆::*imm^434^* | This work |
| mt102-11GFPγ | *AEP1*::GFPγ-Cd*ARG4*/*aep1*∆::Cm*LEU2* *leu2*∆/*leu2*∆ *his1*∆/*his1*∆ *arg4*∆/*arg4*∆  *URA3*/*ura3*∆::*imm^434^* *IRO1*/*iro1*∆::*imm^434^* | This work |
| mt230-11GFPγ | *PET111*::GFPγ-Cd*ARG4*/*pet111*∆::Cm*LEU2* *leu2*∆/*leu2*∆ *his1*∆/*his1*∆ *arg4*∆/*arg4*∆  *URA3*/*ura3*∆::*imm^434^* *IRO1*/*iro1*∆::*imm^434^* | This work |
| mt1179-11GFPγ | *NUO3*::GFPγ-Cd*ARG4*/*nuo3*∆::Cm*LEU2* *leu2*∆/*leu2*∆ *his1*∆/*his1*∆ *arg4*∆/*arg4*∆  *URA3*/*ura3*∆::*imm^434^* *IRO1*/*iro1*∆::*imm^434^* | This work |
| mt1371-11GFPγ | *COE1*::GFPγ-Cd*ARG4*/*coe1*∆::Cm*LEU2* *leu2*∆/*leu2*∆ *his1*∆/*his1*∆ *arg4*∆/*arg4*∆  *URA3*/*ura3*∆::*imm^434^* *IRO1*/*iro1*∆::*imm^434^* | This work |
| mt2513-11GFPγ | *MNE1*::GFPγ-Cd*ARG4*/*mne1*∆::Cm*LEU2* *leu2*∆/*leu2*∆ *his1*∆/*his1*∆ *arg4*∆/*arg4*∆  *URA3*/*ura3*∆::*imm^434^* *IRO1*/*iro1*∆::*imm^434^* | This work |
| mt2819-15GFPγ | *NUE1*::GFPγ-Cd*ARG4*/*nue1*∆::Cm*LEU2* *leu2*∆/*leu2*∆ *his1*∆/*his1*∆ *arg4*∆/*arg4*∆  *URA3*/*ura3*∆::*imm^434^* *IRO1*/*iro1*∆::*imm^434^* | This work |
| mt4467-11GFPγ | *NUE2*::GFPγ-Cd*ARG4*/*nue2*∆::Cm*LEU2* *leu2*∆/*leu2*∆ *his1*∆/*his1*∆ *arg4*∆/*arg4*∆  *URA3*/*ura3*∆::*imm^434^* *IRO1*/*iro1*∆::*imm^434^* | This work |
| mt5077-11GFPγ | *NUO4*::GFPγ-Cd*ARG4*/*nuo4*∆::Cm*LEU2* *leu2*∆/*leu2*∆ *his1*∆/*his1*∆ *arg4*∆/*arg4*∆  *URA3*/*ura3*∆::*imm^434^* *IRO1*/*iro1*∆::*imm^434^* | This work |
| mt5607-11GFPγ | ORF19.5607::GFPγ-Cd*ARG4*/orf19.5607∆::Cm*LEU2* *leu2*∆/*leu2*∆ *his1*∆/*his1*∆ *arg4*∆/*arg4*∆  *URA3*/*ura3*∆::*imm^434^* *IRO1*/*iro1*∆::*imm^434^* | This work |
| mt6566-11GFPγ | *COE2*::GFPγ-Cd*ARG4*/*coe2*∆::Cm*LEU2* *leu2*∆/*leu2*∆ *his1*∆/*his1*∆ *arg4*∆/*arg4*∆  *URA3*/*ura3*∆::*imm^434^* *IRO1*/*iro1*∆::*imm^434^* | This work |
| mt6918-11GFPγ | *QCE1*::GFPγ-Cd*ARG4*/*qce1*∆::Cm*LEU2* *leu2*∆/*leu2*∆ *his1*∆/*his1*∆ *arg4*∆/*arg4*∆ | This work |

1. McRipley RJ, Erhard PJ, Schwind RA, Whitney RR. 1979. Evaluation of vaginal antifungal formulations in vivo. Postgrad Med J 55:648-52.

2. Noble SM, Johnson AD. 2005. Strains and strategies for large-scale gene deletion studies of the diploid human fungal pathogen Candida albicans. Eukaryot Cell 4:298-309.

3. Noble SM, French S, Kohn LA, Chen V, Johnson AD. 2010. Systematic screens of a Candida albicans homozygous deletion library decouple morphogenetic switching and pathogenicity. Nat Genet 42:590-8.
