# Supplementary material for: Unique, Diverged, and Conserved Mitochondrial Functions Influencing Candida albicans Respiration
Source: mBio. 2019 Jun 25;10(3):e00300-19. doi: 10.1128/mBio.00300-19 (PMC6593398; doi:10.1128/mBio.00300-19)
Supplement: TABLE S2 [file mBio.00300-19-st002.docx]

**Table S2 Primers used for gene deletion, integration mapping and complementation**

| Target gene | Primer name^a^ | Primer Sequence |
| --- | --- | --- |
| orf19.94 | 94-5'frag_fwd | CCTTGTTCCTCCACCATTCT |
|  | 94-5'frag_rev^b^ | cacggcgcgcctagcagcggCGATGATCGGATATGAGGAAA |
|  | 94-3'frag_fwd^b^ | gtcagcggccgcatccctgcCTACCATTTCATCGATGGCT |
|  | 94-3'frag_rev | TGAAGCTGCTCCTCAACAAC |
|  | 94-locuscheck_fwd | TTGTTGGACTGATTCGCACT |
|  | 94-locuscheck_rev | AATCTTACCCATCTGCCCATC |
| *AEP1* | 102-5'frag_fwd | GCATTTCACTGGAAATATTGGTT |
|  | 102-5'frag_rev | cacggcgcgcctagcagcggTTGTATATTTCGCTCTCCACCA |
|  | 102-3'frag_fwd | gtcagcggccgcatccctgcGGCAATTTTCAGCAGAGGAG |
|  | 102-3'frag_rev | CAGATCTCCGCCAAATCTGT |
|  | 102-locuscheck_fwd | TTTTGGCACAATCATTTCTCC |
|  | 102-locuscheck_rev | GTGGAAGCAGTCCACATGAGT |
|  | 102_compl_fwd^c^ | gtacgtgggcccTGGCACAATCATTTCTCCAA |
|  | 102_compl_rev^c^ | gtacgtgggcccTGGAACGATAATGCCAGTGA |
| *PET111* | 230-5'frag_fwd | TTGAGGGGGTTGTCATGAAT |
|  | 230-5'frag_rev | cacggcgcgcctagcagcggTTGGTTGATTGGTTGATTGG |
|  | 230-3'frag_fwd | gtcagcggccgcatccctgcTAGTTTGGTGGGTGAGGTGAG |
|  | 230-3'frag_rev | GGACACATACAAGTGTGGGAAA |
|  | 230-locuscheck_fwd | TGCCAAAATGTCGCATCATA |
|  | 230-locuscheck_rev | TTACATACCATTGATTCCCACA |
|  | 230_compl_fwd2 | gtacgtgggcccTTGAGGGGGTTGTCATGAAT |
|  | 230_compl_rev2 | gtacgtgggcccCTCACCTCACCCACCAAACT |
|  | 230_int_rev | TTCTGGCGATAACAGGTTCC |
| orf19.265 | 265-5'frag_fwd | GGGAGTGAGAAAAGAAAAAGAATG |
|  | 265-5'frag_rev | cacggcgcgcctagcagcggAAGGAAGGGATTTCTAAGAGAGGA |
|  | 265-3'frag_fwd | gtcagcggccgcatccctgcCATCATGTCCCTCACTGTCACT |
|  | 265-3'frag_rev | AATAGCATCCACACCAATTCCT |
|  | 265-locuscheck_fwd | ATCAAATGAAAATCGGGAAGAA |
|  | 265-locuscheck_rev | ATGGAGAAGATGAGGAAGACGA |
| orf19.411 | 411-5'frag_fwd | ATCGGACTTGACGCACTACC |
|  | 411-5'frag_rev | cacggcgcgcctagcagcggGAAAAAGAACTGTTGATGATTTGC |
|  | 411-3'frag_fwd | gtcagcggccgcatccctgcTTTTTGGAACGGTTGAGGAT |
|  | 411-3'frag_rev | GATGATGGTACGGCCAAAGT |
|  | 411-locuscheck_fwd | ATCTGATACACGCCACCACA |
|  | 411-locuscheck_rev | TTTGTTCGTTCGTTCGTTCA |
| orf19.527 | 527-5'frag_fwd | AGTTATCTTCGGTTGAAACATCA |
|  | 527-5'frag_rev | cacggcgcgcctagcagcggTGTACAACTTGTTGTGGATTATATTGC |
|  | 527-3'frag_fwd | gtcagcggccgcatccctgcGTAAATTGCTGTAATTGGTAATACGTTG |
|  | 527-3'frag_rev | AGGTTTGGCTTTGTCATTGG |
|  | 527_locuscheck2_fwd | ATGCCACTGAATTGTCCTCAC |
|  | 527_locuscheck2_rev | GTCAAGCTTTGGTGAAGATGC |
| *FGR39* | 679-5'frag_fwd | AACTGCCATACGTGTGCTTG |
|  | 679-5'frag_rev | cacggcgcgcctagcagcggTGCTGCTCGAGATGTGACTC |
|  | 679-3'frag_fwd | gtcagcggccgcatccctgcCAAGACCTCAATGGGAGGAA |
|  | 679-3'frag_rev | TTGTTTTGGGTCGGATTCAT |
|  | 679-locuscheck_fwd | TGGCATTGTCTTTGGATGAA |
|  | 679-locuscheck_rev | TTGGCCTTTTGGGTTTTATG |
| *AGA1* | 935-5'frag_fwd | ACTCATTGAATTTCCCTGCT |
|  | 935-5'frag_rev | cacggcgcgcctagcagcggGACCAGCAAAGAAACAAAGGC |
|  | 935-3'frag_fwd | gtcagcggccgcatccctgcCCATGCTAGAATTCAATGGAGC |
|  | 935-3'frag_rev | AATTCTAAGCCGTCTATTTCAACCT |
|  | 935_locuscheck2_fwd | TCACAAACCAAACGTCAATCA |
|  | 935_locuscheck2_rev | GCAATCAAATCCCAATCAATG |
| *NUO3* | 1179-5'frag_fwd | CTGGGTCAGTACCATACTGCAA |
|  | 1179-5'frag_rev | cacggcgcgcctagcagcggTTTGGAAAGTACTGTGCTGTGG |
|  | 1179-3'frag_fwd | gtcagcggccgcatccctgcCCCAGCTTCTTGATAAATTTGG |
|  | 1179-3'frag_rev | AAGAAAGTGGTGACGACCTGAT |
|  | 1179-locuscheck_fwd | CGCCAGCAGTCAAATTGATA |
|  | 1179-locuscheck_rev | TTGGAGTTTGGCATGATTGA |
|  | 1179compl_fwd | tttccgcggACAGGAAGCAATTCCCTGGT |
|  | 1179compl_rev | tttccgcggCAATAACGACGACGACGATG |
| orf19.1287 | 1287-5'frag_fwd | TCCCATACAATTCGGACTAGAGA |
|  | 1287-5'frag_rev | cacggcgcgcctagcagcggTTGTTGTTGTGACATTATGCAA |
|  | 1287-3'frag_fwd | gtcagcggccgcatccctgcCAAGAGGGGTTTTATCCCAAG |
|  | 1287-3'frag_rev | CCGATATTCAACCGAATCACT |
|  | 1287_locuscheck2_fwd | TTCCTTATTCTTTTATATTCCAGTTCA |
|  | 1287_locuscheck2_rev | TCCCTCTCCTCACATCCTTC |
| orf19.1344 | 1344-5'frag_fwd | TCAAAGAGTTTGTCGCGATG |
|  | 1344-5'frag_rev | cacggcgcgcctagcagcggAAGATGCGTCGTGTATGTGC |
|  | 1344-3'frag_fwd | gtcagcggccgcatccctgcTGGCTGAATTTGCTGATTTG |
|  | 1344-3'frag_rev | TCGATGTCGAGCAGTTTCAC |
|  | 1344-locuscheck_fwd | TGAAAGAGTGATGTGGCTGTG |
|  | 1344-locuscheck_rev | GGCAAAGGGAGAGAAATTACG |
| *COE1* | 1371-5'frag_fwd | CAGCGTATCTGGAACCATCA |
|  | 1371-5'frag_rev | cacggcgcgcctagcagcggCCCATTTCCCGAAAGGATAC |
|  | 1371-3'frag_fwd | gtcagcggccgcatccctgcTCGAAAACTGGTTGTCTCCT |
|  | 1371-3'frag_rev | AGGTGTTGTTGTTGGTGCTG |
|  | 1371-locuscheck_fwd | GAGGCTGCAAAATGGATACAG |
|  | 1371-locuscheck_rev | CCTTGCAAAGTTCAGGAATCA |
|  | 1371compl_fwd | tttccgcggAAGTAGGGGACACCGTAGCC |
|  | 1371compl_rev | tttccgcggAGGTGTTGTTGTTGGTGCTG |
| orf19.1748 | 1748-5'frag_fwd | CCTCCGTCTAACGCAAATTC |
|  | 1748-5'frag_rev | cacggcgcgcctagcagcggAATCTAGGGTGGTGGGTGGT |
|  | 1748-3'frag_fwd | gtcagcggccgcatccctgcCCATCTCATGTAGGGATAATTGG |
|  | 1748-3'frag_rev | AGATCAAGATCCCCAGAACG |
|  | 1748-locuscheck_fwd | CCTATTCCGCTTTTCATTCC |
|  | 1748-locuscheck_rev | TATGACCGTGATCGTGAGGA |
| orf19.1873 | 1873-5'frag_fwd | CCCCTTTCTATTTGACCAACAC |
|  | 1873-5'frag_rev | cacggcgcgcctagcagcggCAGTGATCTTGCAAACATTGTG |
|  | 1873-3'frag_fwd | gtcagcggccgcatccctgcAAGTGTTTGATGGTGGGAGAGT |
|  | 1873-3'frag_rev | GAGCACTTGCATTGACAAAGTC |
|  | 1873_locus_check_fwd | aaaaacattgagtgcaaaatgc |
|  | 1873_locus_check_rev | aaccccaatagccagatgagt |
| orf19.1913 | 1913-5'frag_fwd | TGTTCCCACTTGCGTTACTG |
|  | 1913-5'frag_rev | cacggcgcgcctagcagcggTGTGTTGCGACCAAGAATGT |
|  | 1913-3'frag_fwd | gtcagcggccgcatccctgcCCGCGAGCTATCACAATTAATAC |
|  | 1913-3'frag_rev | GCTGTTGTTTGGGTGTCTAACTC |
|  | 1913-locuscheck_fwd | TGGTTGTCTTTGCACTGGAG |
|  | 1913-locuscheck_rev | GCGTGACTTGACTGGCACTA |
| *MNE1* | 2513-5'frag_fwd | GACAAAGTGGGTTGTTGAAGG |
|  | 2513-5'frag_rev | cacggcgcgcctagcagcggCTAGTTTCTTCGGGATTGCAC |
|  | 2513-3'frag_fwd | gtcagcggccgcatccctgcTTGATAATGACTTGTATGAATGGTGA |
|  | 2513-3'frag_rev | CATTTGAATTTTGCCAATTGAT |
|  | 2513-locuscheck_fwd | TTAATTTGGCATGTTTTGATCG |
|  | 2513-locuscheck_rev | AGGATTATCAATGGGTTTTAATTG |
|  | 2513_compl_fwd2 | gtacgtgggcccTGGCATGTTTTGATCGTTGT |
|  | 2513_compl_rev2 | gtacgtgggcccTATACCTTCGGCTGCTCCTG |
| orf19.2650 | 2650-5'frag_fwd | GGGTGTTTGAGTGTGAGTGTGT |
|  | 2650-5'frag_rev | cacggcgcgcctagcagcggGCTGATGATGCTGATGTTGAAT |
|  | 2650-3'frag_fwd | gtcagcggccgcatccctgcTTTGGTCAAGATGTTGTTACCG |
|  | 2650-3'frag_rev | TTGAAAAGGAGAGTTGGAATTGA |
|  | 2650-locuscheck_fwd | GCGTAAATTGAGGTCGTGTGT |
|  | 2650-locuscheck_rev | CTTCACCATTAGATGCCCAAC |
|  | 2650_int_rev | CATTTCCCTCATTTCCCTCA |
| *NUE1* | 2819-5'frag_fwd | CGAAGTGCATCTTCTCTTTTCAT |
|  | 2819-5'frag_rev | cacggcgcgcctagcagcggAGAGATTGGTTTCAGACCTGTTG |
|  | 2819-3'frag_fwd | gtcagcggccgcatccctgcTGATGATGATGATGACGAGGT |
|  | 2819-3'frag_rev | CTGCAGCAGAACAATCTGACA |
|  | 2819-locuscheck_fwd | TCAGAAACTGGTGGTGTTGC |
|  | 2819-locuscheck_rev | CATCGAAAAAGGGATCGGTA |
|  | 2819compl_fwd | tttccgcggAACGACGACGGAGTTTCCTT |
|  | 2819compl_rev | tttccgcggCATCGAAAAAGGGATCGGTA |
| orf19.3563 | 3563-5'frag_fwd | TCGAACAACCCATCAATTACAA |
|  | 3563-5'frag_rev | cacggcgcgcctagcagcggTGAAGACGATCGTTGTCGAGT |
|  | 3563-3'frag_fwd | gtcagcggccgcatccctgcTTTTTCAGTTCTTCTTTGATGTGT |
|  | 3563-3'frag_rev | TGGATCCATCCAAGAGATTAAG |
|  | 3563-locuscheck_fwd | TACACGCACAAACACCAACA |
|  | 3563-locuscheck_rev | AATGGATGTTTCCCATCTGA |
| *GOA1* | goa1-5'frag_fwd | TGATGGATCTCGGAATTTTTG |
|  | goa1-5'frag_rev | cacggcgcgcctagcagcggGCACCAGGGTTTGTGATTCT |
|  | goa1_3'frag_fwd2 | gtcagcggccgcatccctgcTTGGGTGTCGTGCTGATTTA |
|  | goa1_3'frag_rev2 | AAGCATAGGGAAAACGCAGA |
|  | goa1-locuscheck_fwd | GTTCCAAACTCTGCCTTTGC |
|  | goa1-locuscheck_rev | CCATGACGAAATGACGTTGA |
|  | goa1_compl_fwd | gtacgtgggcccTGCAGCGGTGAAGATAGATG |
|  | goa1_compl_rev | gtacgtgggcccAAGCATAGGGAAAACGCAGA |
| *NUE2* | 4467-5'frag_fwd | TGAAGTGGCTGGTGTAATGG |
|  | 4467-5'frag_rev | cacggcgcgcctagcagcggTGGTGGGTGGGATATATCAAG |
|  | 4467-3'frag_fwd | gtcagcggccgcatccctgcAAATTCTCTGCCTCCACACG |
|  | 4467-3'frag_rev | CCTGGTGGTTGAGGAATAGTT |
|  | 4467-locuscheck_fwd | TTGATGCTGCTGCTGAGTG |
|  | 4467-locuscheck_rev | TGCTGACATGGTGGAATCAA |
|  | 4467_compl_fwd2 | gtacgtgggcccGTCGACAGGGAGCAGGTAAG |
|  | 4467_compl_rev2 | gtacgtgggcccCGTGTGGAGGCAGAGAATTT |
| orf19.4553 | 4553-5'frag_fwd | TGTCAATATCGAAAGGTTTCCTG |
|  | 4553-5'frag_rev | cacggcgcgcctagcagcggAGGAGGAATGGCAAATGAAA |
|  | 4553-3'frag_fwd | gtcagcggccgcatccctgcTTGAATGTTTGGAATGTCTGTTATATACG |
|  | 4553-3'frag_rev | CCCTCACACACAACAACAACC |
|  | 4553-locuscheck_fwd | AGTAAACCTGCCACCCATCTT |
|  | 4553-locuscheck_rev | TCTTGTGGTGGCACAGTTTC |
|  | 4553_int_rev | GTGGAGGTGGTGAGGAAGAA |
| orf19.4734 | 4734-5'frag_fwd | TTCTAGGAACGTTGGTAAAGTTAATC |
|  | 4734-5'frag_rev | cacggcgcgcctagcagcggGCACACACACAGACACAAAGG |
|  | 4734-3'frag_fwd | gtcagcggccgcatccctgcCCAACTCGAGCTCGCTTATT |
|  | 4734-3'frag_rev | TGGTTGAAATGCATCGAGAA |
|  | 4734-locuscheck_fwd | AAGAGCTGTACCTTGTAGTG |
|  | 4734-locuscheck_rev | CCTTCAATTGTCCGGTGATT |
| orf19.4795 | 4795-5'frag_fwd | ATGCAGATGCATTACCAGCTC |
|  | 4795-5'frag_rev | cacggcgcgcctagcagcggGGGTCATGCTCAATATGCAGT |
|  | 4795-3'frag_fwd | gtcagcggccgcatccctgcAATTGTCCTGGTGGCAAAAA |
|  | 4795-3'frag_rev | TTGCAACATTTGCATTGATT |
|  | 4795-locuscheck_fwd | TCATCTCAATTTGGTTGAGTGG |
|  | 4795-locuscheck_rev | GCCAGTCGATGTGATGATTG |
|  | 4795_int_rev | ACATGAAAAGCAGGGACCAA |
| orf19.4895 | 4895-5'frag_fwd | CAAGCAATGTTCAACGGAAGT |
|  | 4895-5'frag_rev | cacggcgcgcctagcagcggTGAAACCATGCATCACCAAA |
|  | 4895-3'frag_fwd | gtcagcggccgcatccctgcACAACCATGGGTTGGAAAAG |
|  | 4895-3'frag_rev | CCCTGCGAAATCTCATTTGT |
|  | 4895-locuscheck_fwd | GAGGAGGAGGAGGAAGAGTCA |
|  | 4895-locuscheck_rev | TATCAGATGGCGGTTTTCAAT |
| *NUO4* | 5077_5'frag_fwd | CACCGCATTCAGTTCTGTTG |
|  | 5077_5'frag_rev | cacggcgcgcctagcagcggACAGTTCATTGAGACTTCAACCA |
|  | 5077_3'frag_fwd | gtcagcggccgcatccctgcGAGGGGATTCAAATCGAACA |
|  | 5077_3'frag_rev | CAGTTCTCGATCGCAAAGGT |
|  | 5077_locus_check_fwd | AACCGGCAATGTTTTGCTAC |
|  | 5077_locus_check_rev | ATTCTGAATGTTGGGGGTGA |
|  | 5077_compl_fwd2 | gtacgtgggcccAACCGGCAATGTTTTGCTAC |
|  | 5077_compl_rev2 | gtacgtgggcccCAGTTCTCGATCGCAAAGGT |
| orf19.5607 | 5607-5'frag_fwd | GGCAAACATTTTGGATTGCTA |
|  | 5607-5'frag_rev | cacggcgcgcctagcagcggAAAACAACAACCAACGACGAG |
|  | 5607-3'frag_fwd | gtcagcggccgcatccctgcCGGTTTTTCGATTTTGTTTTG |
|  | 5607-3'frag_rev | TGGGATTTTTAAAGCTACTTGG |
|  | 5607_locuscheck2_fwd | ACCCTGGAGCTGTGATGAAC |
|  | 5607-locuscheck_rev | ATCCAGGGGTGAATTTGGAT |
|  | 5607_compl_fwd | gtacgtgggcccGTTGTTGCGGTGTTTGCTTAT |
|  | 5607_coml_rev | gtacgtgggcccGAAGTTGGTGGGAATCAAGAA |
| *COE2* | 6566-5'frag_fwd | GGTTTTCAAAGATGCTGAAGTTG |
|  | 6566-5'frag_rev | cacggcgcgcctagcagcggTCCTGTCAATAAACCAAATCACC |
|  | 6566-3'frag_fwd | gtcagcggccgcatccctgcTCCCTAGCTTACAATTGCTTGA |
|  | 6566-3'frag_rev | TCACAAAATGTCTGCCCCTA |
|  | 6566-locuscheck_fwd | GACCGGGTGGCTATGTTAGA |
|  | 6566-locuscheck_rev | TCAGGATATTGCTGTTGTTGG |
|  | 6566compl_fwd | tttccgcggTATGGGTGGTATTATAGTTATAGTAATAGTTC |
|  | 6566compl_rev | tttccgcggGAACTTGACGTTTAAAAGTATGTGGTAG |
| orf19.6853 | 6853-5'frag_fwd | GGAAACAGCGAGTTTGTTGG |
|  | 6853-5'frag_rev | cacggcgcgcctagcagcggTCAACCGGCACAGTTAGAAA |
|  | 6853-3'frag_fwd | gtcagcggccgcatccctgcTCTAACAAAGCGTGTATTTGGTT |
|  | 6853-3'frag_rev | TTTCACTGATAACCCCTCGATT |
|  | 6853-locuscheck_fwd | CCCCCATTCTGATTGAAGAA |
|  | 6853-locuscheck_rev | GTGCAAGGCAGAAAAAGAGG |
| *QCE1* | 6918-5'frag_fwd | TTGATGATCTGCAAGGATTGATA |
|  | 6918-5'frag_rev | cacggcgcgcctagcagcggTGCCTTTTATTTGCCCTTTTTAT |
|  | 6918-3'frag_fwd | gtcagcggccgcatccctgcCGACAAAGTGCTGATAAAAGAGG |
|  | 6918-3'frag_rev | TACTCATAACCCTCACGCCTAAA |
|  | 6918-locuscheck_fwd | TTTGGCAATGATGCTGATGT |
|  | 6918-locuscheck_rev | CGTGAAGTTTCTGCGTCAAA |
|  | 6918_compl_fwd | gtacgtgggcccTTGAAATTCCAGATAAGGGTTGTT |
|  | 6918_compl_rev | gtacgtgggcccACCATCATCAGTCTTGTCATCAGT |
| Cm*LEU2* | CmLEU2fusion_fwd | ccgctgctaggcgcgccgtgTTTGTGTTGTTATGTGTAATTATCGTG |
|  | CmLeu2fusion_rev | gcagggatgcggccgctgacGCCAATCAGAATCAAGTGAGG |
|  | CmLEU2_locuscheck_fwd | CCAGATACCAACACCGCTTT |
|  | CmLEU2_locuscheck_rev | AGTGGCAGCTTCAATTGCTT |
| Cd*HIS1* | CdHIS1fusion_fwd | ccgctgctaggcgcgccgtgCTCAACTGGGGGTTTAACAAA |
|  | CdHIS1fusion_rev | gcagggatgcggccgctgacAAAGGTGTGTACATCAAGGTGGT |
|  | CdHIS1_locuscheck_fwd | TGTTTTAGCGGCTCAGGAAT |
|  | CdHIS1_locuscheck_rev | GATCGGGCAAATGATTGACT |

a. Locus specific sequences flanking the ORF were generated with primer pairs orf-5’frag_fwd/orf-5’frag_rev and orf-3’frag_fwd/orf-3’frag_rev and fused with selection markers Cm*LEU2* or Cd*HIS1* generated with primer pairs CmLEU2fusion_fwd/ CmLeu2fusion_rev or CdHIS1fusion_fwd/CdHIS1fusion_rev, respectively. Linkage of the marker gene in transformed cells at the locus of interest was verified with the relevant “locuscheck” primers. Absence of an orf in putative null mutants was verified using orf-5’frag_fwd and orf-3’frag_rev primers to amplify disrupted and potential wild-type alleles. However, for longer ORFs internal primers were used in conjunction with orf-locuscheck_rev primers.

b. Lower case letters in the orf-5’frag_rev and orf-3’frag_fwd primers designate inserted sequences complementary to those flanking the marker genes {Noble, 2005 #3587}.

c. Lower case letters in the “orf-compl” primers designated added *Sac*II or *Apa*I recognition sites.
