# Supplementary material for: Unique, Diverged, and Conserved Mitochondrial Functions Influencing Candida albicans Respiration
Source: mBio. 2019 Jun 25;10(3):e00300-19. doi: 10.1128/mBio.00300-19 (PMC6593398; doi:10.1128/mBio.00300-19)
Supplement: TABLE S3 [file mBio.00300-19-st003.docx]

**Table S3. Primers for plasmid pleuARGleu construction and verification of integration at *LEU2*.**

| **Target gene** | **Primer name** | **Primer sequence** |
| --- | --- | --- |
|  | pUC19_AscI_fwd^a^ | atatatggcgcgccCAAAGGCGGTAATACGGTTATC |
|  | pUC19_AscI_rev^a^ | atatatggcgcgccCGGTATTTTCTCCTTACGCATC |
| Cd*ARG4* | CdARG4rescue_fwd | ccgctgctaggcTcgccgtgTGTTCGACCTCCACCAATCT |
|  | CdARG4rescue_rev | tgggcccgcggccgccggctCATCCCAAAGGCGATACAAT |
| Ca*LEU2* | LEU2-5'frag_fwd^a^ | atatatggcgcgccAAGCCGAAGTCGACTATGTC |
|  | LEU2-5'frag_rev^b^ | cacggcgAgcctagcagcggGGATATTGGTTTTAAAAGAAAGG |
|  | LEU2-3'frag_fwd^c^ | agccggcggccgcgggcccaACAGTATATACAGTAGTTAGC |
|  | LEU2-3'frag_rev^a^ | atatatggcgcgccTGACAAATGAATTCAGTCAG |
|  | LEU2-5'locuscheck_fwd | GCTTTGAGTTCTGGGTCAGC |
| Cd*ARG4* | LEU2-5'locuscheck_rev | AAAGATTGGTGGAGGTCGAA |
|  | LEU2-locuscheck_rev | GACCAGACTCTATCCCAAAG |

a. Lower case indicates added *Asc*I recognition sequence.

b. Lower case indicates inserted sequences complementary to those flanking the 5’-end of Cd*ARG4*.

c. Lower case indicates inserted sequences complementary to those flanking the 3’-end of Cd*ARG4* and containing restriction sites for *Apa*I, *Sac*II, *Not*I, *Eag*I and *Nae*I.
