# Supplementary material for: Unique, Diverged, and Conserved Mitochondrial Functions Influencing Candida albicans Respiration
Source: mBio. 2019 Jun 25;10(3):e00300-19. doi: 10.1128/mBio.00300-19 (PMC6593398; doi:10.1128/mBio.00300-19)
Supplement: TABLE S4 [file mBio.00300-19-st004.docx]

**Table S4. Primers used in restoration of the *ARG*4 locus.**

| **Target gene** | **Primer name** | **Primer sequence** |
| --- | --- | --- |
| Ca*ARG4* | CaARG4restore_fw | TAACTTTGGGTCGTGTACTTTTGA |
|  | CaARG4restore_rev | CAGACGATCTTTACAATGGAAGTG |
|  | CaARG4_5'val_fwd2 | ATCGATTGAGATCGGTATGGTATT |
|  | CaARG4_5'val_rev2 | TGAAACGACCACCCCATAAT |
|  | CaARG4_3'val_fwd2 | AGATGCTATTGGTGGTACTGCT |
|  | CaARG4_3'val_rev2 | CGTTTGGAAGCTGTATATCGTG |
