# Supplementary material for: Unique, Diverged, and Conserved Mitochondrial Functions Influencing Candida albicans Respiration
Source: mBio. 2019 Jun 25;10(3):e00300-19. doi: 10.1128/mBio.00300-19 (PMC6593398; doi:10.1128/mBio.00300-19)
Supplement: TABLE S5 [file mBio.00300-19-st005.docx]

**Table S5. Primers used in construction of GFP tagged genes**

| **Taget gene** | **Primer Name** | **Primer Seq^b^** |
| --- | --- | --- |
| *AEP1*^a^ | 7749_GFP_5'frag_fwd | CTGGCCATTTTTAGCAGAGG |
|  | 7749_GFP_5'frag_rev | gaagcacctgcgccagcaccGTTTTCTAAAACCCTCATCAAATC |
|  | 7749_GFP_3'frag_fwd | ctcgaattcatcgatgatatcagaAGTCGACAGAGTTGGGAGAGA |
|  | 102_GFP_3'frag_rev | TGGAACGATAATGCCAGTGA |
|  | 7749_GFP_5'lc_fwd | CCCGAAACGAAATTGAGAGA |
|  | 7749_GFP_3'lc_rev | CGATTGTGCTGATGGCTTTA |
| *PET111* | 230_GFP_5'frag_fwd | TTCAAGAAATTACGATCAATACAGAA |
|  | 230_GFP_5'frag_rev | gaagcacctgcgccagcaccCCTATTATTATTATCTTTCCATTGATTT |
|  | 230_GFP_3'frag_fwd | ctcgaattcatcgatgatatcagaAGTTTGGTGGGTGAGGTGAG |
|  | 230_3'frag_rev | GGACACATACAAGTGTGGGAAA |
|  | 230_GFP_5'lc_fwd | TTCCTCCTAATAATCAAGGATCAAA |
|  | 230_locuscheck_rev | TTACATACCATTGATTCCCACA |
| *NUO3* | 1179_GFP_5'frag_fwd | CCATCCAAAACACACAACTGC |
|  | 1179_GFP_5'frag_rev | gaagcacctgcgccagcaccTTGATATTTAGCACCAAATTTATCAAG |
|  | 1179_GFP_3'frag_fwd | ctcgaattcatcgatgatatcagaAACAATATACATTTATTCGATTTCAATAC |
|  | 1179_3'frag_rev | AAGAAAGTGGTGACGACCTGAT |
|  | 1179-5'frag_fwd | CTGGGTCAGTACCATACTGCAA |
|  | 1179_locuscheck_rev | TTGGAGTTTGGCATGATTGA |
| *COE1* | 1371_GFP_5'frag_fwd | ATTGATCCACATCGAATCCAA |
|  | 1371_GFP_5'frag_rev2 | gaagcacctgcgccagcaccCATGATTTTTTGATATGAAACAAGT |
|  | 1371_GFP_3'frag_fwd | ctcgaattcatcgatgatatcagaTCGAAAACTGGTTGTCTCCT |
|  | 1371_GFP_3'frag_rev | GGTGTCGTCGTTGTTTTCAA |
|  | 1371_GFP_5'lc_fwd | ATTGGCAGGAGAAGTGATGG |
|  | 1371_locuscheck_rev | CCTTGCAAAGTTCAGGAATCA |
| *MNE1* | 2513_GFP_5'frag_fwd | AATCAATTGGCAAAATTCAAA |
|  | 2513_GFP_5'frag_rev | gaagcacctgcgccagcaccTTGAAATATTTTCATTAAAGTTTCTGA |
|  | 2513_GFP_3'frag_fwd | ctcgaattcatcgatgatatcagaACATCTTATTGGTATATATAGATATAG |
|  | 2513_GFP_3'frag_rev2 | GAAGATGACGACGACGAAGA |
|  | 2513_GFP_5'lc_fwd | AAGGAGTTTTAGATTATGGTGGTCA |
|  | 2513_GFP_3'lc_rev2 | CACAGCACAGCACTTCCTTT |
| *NUE1* | 2819_GFP_5'frag_fwd | TGCCAATGGAACTAATGATG |
|  | 2819_GFP_5'frag_rev | gaagcacctgcgccagcaccTTTCAATCTATTTATTAATCTATTTGG |
|  | 2819_GFP_3'frag_fwd | ctcgaattcatcgatgatatcagaCAAAACTTTCTTGGGTATCGGT |
|  | 2819_GFP_3'frag_rev | CATCGAAAAAGGGATCGGTA |
|  | 2819_GFP_5'lc_fwd | TTGACCAGTTTAGTGGCTATTCC |
|  | 2819_GFP_3'lc_rev | TGACCCAAATAGTTCGATTGGT |
| *NUE2* | 4467_GFP_5'frag_fwd | TCGATTTGATATCCCTTGGTATG |
|  | 4467_GFP_5'frag_rev | gaagcacctgcgccagcaccCTTGGCTAATGGATATAACCATT |
|  | 4467_GFP_3'frag_fwd | ctcgaattcatcgatgatatcagaGACCGGTGTTGTTGTTGTTG |
|  | 4467_GFP_3'frag_rev | CGTGTGGAGGCAGAGAATTT |
|  | 4467_GFP_5'lc_fwd | CCCACTTTTCAATGTTGATCG |
|  | 4467_3’frag_rev | CCTGGTGGTTGAGGAATAGTT |
| *NUO4* | 5077_GFP_5'frag_fwd | CTGGGGAACGTAGCCACTTA |
|  | 5077_GFP_5'frag_rev | gaagcacctgcgccagcaccATTTTCAGCTGGTTGTTGAAG |
|  | 5077_GFP_3'frag_fwd | ctcgaattcatcgatgatatcagaGAGGGGATTCAAATCGAACA |
|  | 5077_3’frag_rev | CAGTTCTCGATCGCAAAGGT |
|  | 5077_GFP_5'lc_fwd | CTGGGGAACGTAGCCACTTA |
|  | 5077_locuscheck_rev | ATTCTGAATGTTGGGGGTGA |
| *COE2* | 6566_GFP_5'frag_fwd | TCAATGATTTGCCAGCTTTG |
|  | 6566_GFP_5'frag_rev | gaagcacctgcgccagcaccCGCTTGTAACACTTGAACATTAATG |
|  | 6566_GFP_3'frag_fwd | ctcgaattcatcgatgatatcagaTCCCTAGCTTACAATTGCTTGA |
|  | 6566-3'frag_rev | TCACAAAATGTCTGCCCCTA |
|  | 6566_GFP_5'lc_fwd | CTGGACACAACCCAGTTATCAA |
|  | 6566-locuscheck_rev | TCAGGATATTGCTGTTGTTGG |
| *QCE1* | 6918_GFP_5'frag_fwd | CAACAACAACAGGAGGAGGAG |
|  | 6918_GFP_5'frag_rev | gaagcacctgcgccagcaccTGGTAATAAATATAATCCTCTTTTA |
|  | 6918_GFP_3'frag_fwd | ctcgaattcatcgatgatatcagaTTCATGAGTATTTCCCCCAGA |
|  | 6918-3'frag_rev | TACTCATAACCCTCACGCCTAAA |
|  | 6918_GFP_5'lc_fwd | TCCTGCCTGATGATTCAAAA |
|  | 6918-locuscheck_rev | CGTGAAGTTTCTGCGTCAAA |
| COX8 | COX8_GFP_5'frag_fwd | CCAGAACCCTTCCATCAAGA |
|  | COX8_GFP_5'frag_rev | gaagcacctgcgccagcaccTTCAGTTTTGTTGAAAGCACCA |
|  | COX8_GFP_3'frag_fwd | ctcgaattcatcgatgatatcagaATGTTTAATCATTAACCAACTGATGTT |
|  | COX8_GFP_3'frag_rev | CTGGTCAAACGCATTTTTCA |
|  | COX8_GFP_5'lc_fwd | GCAAAAATCGCACGACCTAT |
|  | COX8_GFP_3'lc_rev | CAAGTTTGGCCATTCCATCT |
| GOA1 | GOA1-GFP_5'frag_fwd | TCTACTGCGGCCGCTGGGTTACCACAAACGTGAA |
|  | GOA1-GFP_5'frag_rev | gaagcacctgcgccagcaccATTCTGATTTTCTTTGTTGAGAGATT |
|  | GOA1-GFP_3'frag_fwd | ctcgaattcatcgatgatatcagaCGTATTCCGACTAGGAATGCAA |
|  | GOA1-GFP_3'frag_rev | TCTACTGCGGCCGCTCTCAAACAATGCCAACCAC |
|  | GOA1-GFP_5'locuscheck_fwd | TATACTGGAGCATGGCGTTG |
|  | GOA1-GFP_3'locuscheck_rev | CGGTGTTTTGGGTACCTCTC |
|  |  |  |
| GFPγ | GFPgamma_lc_rev^c^ | CCTTCACCGGAGACAGAAAA |
| ARG4 | CaARG4_lc_fwd^d^ | GATGCTCTTGGTGGTACTGCT |

a. orf19.7749 is allelic with orf19.102, but due to extensive heterozygosity at the locus, allele-specific primers were required.

b. Nucleotide sequences complementary to pFA-GFPγ-ARG4 are designated by lower case letters {Schaub, 2006 #3890;Zhang, 2011 #4872}.

c. GFPgamma_lc_rev was used to test 5'linkage of integration events at each locus.

d. CaARG4_lc_fwd was used to test 3'linkage of integration events at each locus.
