# Supplementary material for: Unique, Diverged, and Conserved Mitochondrial Functions Influencing Candida albicans Respiration
Source: mBio. 2019 Jun 25;10(3):e00300-19. doi: 10.1128/mBio.00300-19 (PMC6593398; doi:10.1128/mBio.00300-19)
Supplement: TEXT S1 [file mBio.00300-19-s0001.docx]

**Methods S1.**

**Strain constructions.** Deletion mutants were constructed in *C. albicans* strain SN152 essentially as described by Noble and Johnson (1). Cm*LEU2* and Cd*HIS1* were used as selectable markers and amplified from plasmids pSN40 and pSN52 respectively (1). However, only a portion of the inserts were amplified to eliminate irrelevant flanking genes and sequences. (Strain SN152 and plasmids pSN40 and pSN52 were generously provided by Suzanne Noble.) Integration of transforming DNA at the target site, as well as loss of the open reading frame, were verified by PCR (1). Null mutants were converted to Arg^+^ by transformation with *ARG4* DNA amplified from *C. albicans* strain SC5314

Complementation tests employed plasmid pleuARGleu for cloning of the gene of interest and subsequent integration at the *LEU2* locus. Plasmid pleuARGleu was constructed by PCR fusion of DNA fragments containing nucleotides -341 to -8 of Ca*LEU2* and +1123 to +1462 of Ca*LEU2* with nucleotides -749 to +1525 of Cd*ARG4*. The primers incorporated a non-template *Asc*I recognition sequence at the 5’ and 3’ends of the fusion product, as well as recognition sites for *Apa*I, *Sac*II, *Not*I, *Eag*I and *Nae*I between the 3’-end of Cd*ARG4* and the 3’-end fragment of Ca*LEU2*. These sites allow cloning of most any *C. albicans* ORF. The fusion product was cloned into the *Asc*I site of plasmid pUC19-AscI. Plasmid pUCAscI was constructed from pUC19 by PCR amplification with primers pUC19-AscI_fwd and pUC19-AscI_rev followed by restriction enzyme digestion with *Asc*I and ligation. Genes of interest were PCR amplified from genomic DNA of *C. albicans* strain SC5314 with *Apa*I or *Sac*II sites added at each end and cloned into the like sites of pleuARGleu. The resulting plasmids were digested with *Asc*I and used for transformation of the relevant null mutant. Integration at the *LEU2* locus was verified by PCR.

Strains containing the gene of interest fused to GFP were produced using the fusion PCR method described for construction of deletion mutants. Regions of the gene of interest 150-350 nucleotides in length were amplified from the 3’-end of the coding region and immediately downstream of the coding region using strain SC5314 genomic DNA as template. Sequences complementary to the pFA cassette (2) were incorporated into the amplification primers and used to amplify the GFPγ *ARG4* insert of plasmid pFA-GFPγ-ARG4 (3). (Plasmid pFA-GFPγ-ARG4 was kindly provided by J. Konopka.) GFP was fused to the final amino acid codon of the gene of interest. The amplified fusion products were used to transform the corresponding heterozygous deletion mutants. The only exception was GFPγ fused to *COX8*, which was integrated into strain SN152. Integration into the locus of interest was verified by PCR.

Transformations were performed as in (4) and transformed cells were selected on SC drop-out medium. All primers used in construction of the strains are listed in Tables S2 through S5. Sequence analyses were performed in DNA Strider (5) and primers were designed in Primer3 (6).

**Isolation of mitochondria.** Mitochondria were isolated by a modification of previously described procedures (7). Cells cultured to stationary phase in 100 ml YPD medium at 30^0^C were collected by centrifugation for 5 min at 1,500 x g. The cells were washed with 10 ml H_2_O, suspended in 10 ml of 100 mM Tris, pH 9.4, 10 mM DTT and incubated for 15 min at 30^0^C. Following centrifugation, the cell pellet was suspended in 10 ml 1 M sorbitol, 20 mM MOPS, pH 7.4. Cells were converted to spheroplasts by addition of Zymolyase 100T, 2.5 mg in 250 µl of 50% glycerol (8), and incubating 60 min at 30^0^C with gentle shaking. Spheroplasts were collected by centrifugation, 5 min, 1500 x g, and washed with 10 ml 1 M sorbitol, 20 mM MOPS, pH 7.4. Samples and solutions were maintained at 4^0^C in all subsequent steps. The washed spheroplasts were suspended in 10 ml 0.6 M sorbitol, 20 mM MOPS, pH 7.4, 2 mM MgCl_2_, 1 mM EGTA, containing HALT protease inhibitor cocktail (Pierce). Lysis of the spheroplasts was achieved by a single passage though an Aminco pressure cell at 1,000 psi (9). The resulting lysate was centrifuged 5 min at 1,500 x g. The supernatant was retained and the pellet was washed once with 5 ml of 0.6 M sorbitol solution. The second supernatant was combined with the first to yield the total lysate. The total lysate was centrifuged 10 min at 12,000 x g producing a crude mitochondrial pellet. This pellet was suspended in 5 ml 0.6 M sorbitol solution and dispersed by ten strokes in a Dounce homogenizer. Following centrifugation for 5 min at 1,500 x g, the supernatant was recovered and centrifuged 15 min at 12,000 x g. The pellet of washed mitochondria was suspended in 2 ml 0.6 M sorbitol solution, homogenized and layered on a Percoll step gradient (7). The gradient consisted 5 ml of 23% Percoll layered on 2.5 ml of 40% Percoll, both in 0.6 M sorbitol, 20 mM MOPS, pH 7.4, 2 mM MgCl_2_, 1 mM EGTA. The gradient was centrifuged 20 min at 12,000 x g. Material banding at the 40% Percoll interface was recovered, mixed with five volumes of 0.6 M sorbitol solution, and centrifuged 10 min at 17,000 x g. The supernatant was carefully removed from the loose pellet, which was suspended in 5 ml 0.6 M sorbitol solution and centrifuged 10 min at 7,600 x g. The resulting purified mitochondria were suspended in 2 ml 0.6 M sorbitol solution. Twenty µl of the suspension was mixed with 1 µl of 1% Triton X-100 and the solubilized protein content was determined using the Bradford assay (10). The mitochondrial suspension was divided into 500 µg portions, centrifuged 10 min at 12,000 x g, and the resulting pellets were frozen in dry ice-ethanol and stored at -80^0^C.

**Confocal microscopy.** GFP-tagged strains cultured to stationary phase in YPD medium at 30^0^C were inoculated into fresh YPD (1 x 10^6^ cells/ml) and incubated 3-4 h at 30^0^C with vigorous aeration. MitoTracker Red CMXRos (Molecular Probes) (250 nM) was added. After 30 min cells were collected by gentle centrifugation (1.5 min at 700 x g) to avoid mitochondrial fragmentation (11), washed twice with 1 volume of pre-warmed YPD, suspended in 1 volume pre-warmed YPD, and incubated 15 min at 30^0^C with aeration. Methanol free formaldehyde (4%) was added. After 30 min, cells were centrifuged, washed with post-fix solution (10 mM Tris, pH 8, 135 mM NH_4_Cl, 0.1% Triton X-100), suspended in post-fix solution, and incubated at 30^0^C. Cells were collected after 30 min, suspended in fresh post-fix solution, mounted on concanavalin A coated coverslips, and the mounts sealed with paraffin. Samples were protected from light during and subsequent to dye labeling.

For unfixed samples, cells were harvested and washed twice with 10 mM Tris, pH 7.5, 100 mM glucose, 85 mM NaCl after MitoTracker labeling. Washed cells were suspended in Tris-glucose-NaCl solution and mounted as for fixed samples. The inclusion of glucose was necessary to prevent the mitochondria from loosing their tubular morphology.

Images were acquired using a Zeiss LSM510 Meta confocal laser scanning microscope equipped with a Plan-Apochromat 100X/1.4 oil objective. For GFP imaging the sample was exited with the 488 nm line of an argon laser and fluorescence emission was collected with a 500-550 nm bandpass filter. MitoTracker Red CMXRos imaging used 543 nm illumination from a helium neon laser and emission detection with a 560 nm longpass filter. Samples were sequentially scanned and imaged at 3x zoom (0.029  pixels) using a pinhole diameter of 1 Airy unit.

**Literature cited.**

1. Noble SM, Johnson AD. 2005. Strains and strategies for large-scale gene deletion studies of the diploid human fungal pathogen Candida albicans. Eukaryot Cell 4:298-309.

2. Schaub Y, Dunkler A, Walther A, Wendland J. 2006. New pFA-cassettes for PCR-based gene manipulation in Candida albicans. J Basic Microbiol 46:416-29.

3. Zhang C, Konopka JB. 2011. A photostable green fluorescent protein variant for analysis of protein localization in Candida albicans. Eukaryot Cell 9:224-6.

4. Walther A, Wendland J. 2003. An improved transformation protocol for the human fungal pathogen Candida albicans. Curr Genet 42:339-43.

5. Marck C. 1988. "DNA Strider": a C program for the fast analysis of DNA and protein sequences on the Apple Macintosh family of computers. Nucl Acids Res 16:1829-1836.

6. Untergasser A, Cutcutache I, Koressaar T, Ye J, Faircloth BC, Remm M, Rozen SG. 2012. Primer3--new capabilities and interfaces. Nucleic Acids Res 40:e115.

7. Sims NR, Anderson MF. 2008. Isolation of mitochondria from rat brain using Percoll density gradient centrifugation. Nat Protoc 3:1228-39.

8. Ovalle R, Lim ST, Holder B, Jue CK, Moore CW, Lipke PN. 1998. A spheroplast rate assay for determination of cell wall integrity in yeast. Yeast 14:1159-66.

9. Linnane AW, Lukins HB. 1975. Isolation of mitochondria and techniques for studying mitochondrial biogenesis in yeasts. Methods Cell Biol 12:285-309.

10. Bradford MM. 1976. A rapid and sensitive method for the quantitation of microgram quantities of protein using the principle of protein-dye binding. Anal Biochem 72:248-254.

11. Swayne TC, Gay AC, Pon LA. 2007. Visualization of mitochondria in budding yeast. Methods Cell Biol 80:591-626.
